# Comparisons of the composition and biogeographic distribution of the bacterial communities occupying South African thermal springs with those inhabiting deep subsurface fracture water

**DOI:** 10.3389/fmicb.2014.00679

**Published:** 2014-12-17

**Authors:** Cara Magnabosco, Memory Tekere, Maggie C. Y. Lau, Borja Linage, Olukayode Kuloyo, Mariana Erasmus, Errol Cason, Esta van Heerden, Gaetan Borgonie, Thomas L. Kieft, Jana Olivier, Tullis C. Onstott

**Affiliations:** ^1^Department of Geosciences, Princeton UniversityPrinceton, NJ, USA; ^2^Department of Environmental Sciences, School of Agriculture and Environmental Sciences, University of South AfricaFlorida, South Africa; ^3^Department of Microbial, Biochemical and Food Biotechnology, University of the Free StateBloemfontein, South Africa; ^4^Extreme Life IsyensyaGentbrugge, Belgium; ^5^Biology Department, New Mexico Institute of Mining and TechnologySocorro, NM, USA

**Keywords:** subsurface, thermal springs, diversity, 16S rRNA gene, V6 hypervariable region

## Abstract

South Africa has numerous thermal springs that represent topographically driven meteoric water migrating along major fracture zones. The temperature (40–70°C) and pH (8–9) of the thermal springs in the Limpopo Province are very similar to those of the low salinity fracture water encountered in the South African mines at depths ranging from 1.0 to 3.1 km. The major cation and anion composition of these thermal springs are very similar to that of the deep fracture water with the exception of the dissolved inorganic carbon and dissolved O_2_, both of which are typically higher in the springs than in the deep fracture water. The *in situ* biological relatedness of such thermal springs and the subsurface fracture fluids that feed them has not previously been evaluated. In this study, we evaluated the microbial diversity of six thermal spring and six subsurface sites in South Africa using high-throughput sequencing of 16S rRNA gene hypervariable regions. Proteobacteria were identified as the dominant phylum within both subsurface and thermal spring environments, but only one genera, *Rheinheimera*, was identified among all samples. Using Morisita similarity indices as a metric for pairwise comparisons between sites, we found that the communities of thermal springs are highly distinct from subsurface datasets. Although the Limpopo thermal springs do not appear to provide a new window for viewing subsurface bacterial communities, we report that the taxonomic compositions of the subsurface sites studied are more similar than previous results would indicate and provide evidence that the microbial communities sampled at depth are more correlated to subsurface conditions than geographical distance.

## Introduction

Although Whitman et al. (1998) estimated that the terrestrial subsurface biosphere comprises 40–50% of the world's biomass; comprehensive surveys of its phylogenetic diversity and distribution are geographically sparse relative to the immense volume the subsurface encompasses. Traditionally, mines and drilling have been used to access the deep biosphere; however, these activities are costly and alternative means of studying the subsurface are desired. Deming and Baross (1993) were among the first to propose that deep-sea hydrothermal vents could provide “windows” to the deep biosphere of the oceanic crust. This was based upon the reasoning that only subsurface hyperthermophiles could survive within the hydrothermal vent fluids and that these fluids are representative of the sub-seafloor ocean crust at ridges and, by extension, the deep ocean crust globally. More recently, serpentinite seeps and springs have become a popular site for viewing the marine subsurface (Schrenk et al., 2010) as well as the terrestrial subsurface biosphere in California (Barnes et al., 1967; Morrill et al., 2013; Suzuki et al., 2013), Canada (Brazelton et al., 2012, 2013; Szponar et al., 2013), Portugal (Marques et al., 2008), Turkey (Hosgörmez, 2007), Yugoslavia, and Oman (Barnes et al., 1978). The reasoning is that the extremely high pH of these fluids (pH 10–12) can only support subsurface alkaliphilic microorganisms and that these fluids are representative of the ultrabasic serpentinite at depth. These springs provide an attractive target for sampling the terrestrial subsurface at lower cost; however, only a handful of terrestrial serpentinite sites are known and studied. In order to obtain a global picture of the deep terrestrial subsurface, more surficial, terrestrial portals need to be studied and the means for identifying which microorganisms are truly “denizens” of the deep as opposed to spring communities merits further investigation (Brazelton et al., 2012).

Thermal springs are widespread across the continents. Thermal water emerging at the surface has been warmed as a result of volcanic activity or deep circulation of groundwater (Todd, 1980). Gravity driven circulation can transport meteoric water to considerable depths, along fracture zones or dykes, before resurfacing at thermal springs. As water penetrates underground, it is believed to warm at a rate of approximately 2–3°C per 100 m (geothermal gradient) (Press and Siever, 1986) and, thus, the temperature of a thermal spring is directly related to both the depth of water penetration and the rate at which it ascends to the surface (Grasby and Hutcheon, 2001).

There are over 90 thermal springs identified within South Africa (Olivier et al., 2011) and, since volcanic activity has not occurred in over 90 million years, these thermal springs have been attributed to the deep circulation of groundwater (Olivier et al., 2008). The bacterial and algal diversity of the springs have been reported by Tekere et al. (2011, 2012) and Jonker et al. (2013) along with geochemical data (Olivier et al., 2008). Additionally, South Africa is home to 1600 mines and 8 of the 10 deepest mines in the world (Kidd Creek Mine and Creighton Mine of Canada are the other two). Extensive studies of the water biodiversity and geochemistry have been reported (Takai et al., 2001; Moser et al., 2003, 2005; Kieft et al., 2005; Gihring et al., 2006; Lin et al., 2006a,b; Borgonie et al., 2011). The thermal springs of the Limpopo Province occur at low elevations, in the Lowveld, whereas the mines used to access the subsurface meteoric water occur at high elevations, in the Highveld mostly. In principle the meteoric water recharging the water in the Highveld and migrating to depth could be the source of thermal spring discharge in the Lowveld on a regional scale. This unique hydrogeological setting and infrastructure in South Africa provides an unprecedented opportunity to compare the microbial communities of deep fracture water microbial communities to those of thermal springs and assess whether the latter provide portals to the deep biosphere. This study is the first multi-site comparison of thermal springs and deep subsurface fracture waters in South Africa to address this question.

## Materials and methods

As a preliminary investigation of the biological connectivity of thermal springs to the subsurface, 6 thermal springs (Eiland, Mphephu, Sagole, Siloam, Souting, Tshipise) and the fracture water from 6 mines, Beatrix gold mine (Be326), Driefontein gold mine (Dr5IPC), Finsch diamond mine (FI88), Masimong gold mine (MM51940), Zondereinde platinum mine (NO14), and Tau Tona gold mine (TT109) were sampled. The thermal springs are distributed over a distance of 120 km in the Limpopo Province of northern South Africa. The mines occur of a distance of 500 km from northern South Africa to central South Africa. Access to these sites is difficult and, therefore, samples for thermal spring and subsurface datasets were collected separately and later combined for the purpose of this study. A more detailed description of sample collection and analysis is provided below.

### Sample collection and DNA extraction

Thermal spring and subsurface samples were collected using separate procedures. Notably, the geochemical descriptions of Mphephu, Sagole, Siloam, and Tshipsie thermal springs have been previously published in Olivier et al. (2011). For both thermal spring and subsurface locations, water quality measurements such as temperature, pH, electrical conductivity (EC), and total dissolved salinity (TDS) were made *in situ* using handheld probes (thermal spring: Mettler Toledo meters; subsurface: Hanna Instruments). Biological samples from thermal springs represent a combination of both the planktonic and biofilm communities, whereas the subsurface samples encapsulate only the planktonic community.

In total, six thermal springs were sampled in the Limpopo province of South Africa, five from locations within the pools where the spring water was emerging from the ground and one, Siloam, from both the pool and from a pipe inserted into the spring source (Tekere et al., 2011, 2012). Analysis for chemical parameters of the thermal spring water was performed at the Institute for Soil, Climate, and Water (Agricultural Research Council; Pretoria, South Africa) and described in Olivier et al. (2011). Water from Eiland and Souting thermal spring were analyzed in parallel with these samples but not included in the Olivier et al. (2011) publication. For each thermal spring, 2 L of water were filtered onto a 1.2-μm pore size nitrate cellulose filter and biofilm samples were collected and centrifuged at 2,000× g for 10 min. Upon collection, samples were placed in cooler boxes without temperature control and transported back to the University of South Africa for subsequent analysis. DNA was extracted from both the filter and cell pellet using the Genomic DNA Tissue Mini-Prep Kit (Zymo Research) with an additional DNA wash step (Tekere et al., 2011). DNA extracts from the biofilm and filter were pooled for each site and sequenced. In order to estimate the minimum depth from which the thermal water had originated, we subtracted the mean annual surface temperature from the *in situ* temperature of the water as an estimate for how much the thermal water had warmed at depth and then divided this value by the geothermal gradient in the region. In this calculation, we assumed that the average annual temperature of the Limpopo province was 20°C and that the geothermal gradient was between 20°C km^−1^ and 30°C km^−1^ (Dhansay et al., 2014).

Access to deep fracture water for sampling was obtained through boreholes located within gold, platinum and diamond mines throughout South Africa that have been drilled by the mines to detect water-filled fractures and, thus, prevent flooding in the mine. At each underground site, the borehole was opened and water was allowed to flow freely for 5 min to flush the borehole of contaminants. Subsurface fracture water and gas was analyzed using previously published methods (Lin et al., 2006a; Onstott et al., 2006; Lollar et al., 2008). An autoclaved, stainless steel manifold was attached to the borehole and connected to a stainless steel casing containing a pleated, 25-cm long Memtrex NY filter (MNY filter; Cat. No. MNY-91-1-AAS or MNY-92-1-AAS, General Electric Co.). Water was allowed to flow at a rate of ~4 L/min through the filter and left for a period of approximately 2 weeks to collect biomass. The volume of water that flowed through the filter was measured with a flow accumulator and the amount of captured biomass was estimated by multiplying this volume by the cell counts (Supplement Table 1). The filters were transported back to Princeton using a MVE ZC 20/3V vapor shipper and stored at −80°C until further processing. The borehole located in the Beatrix gold mine was sampled on two occasions, January 25, 2011 and July 27, 2012, and are reported as Be326_11 and Be326_12, respectively. DNA extraction from the MNY filters was performed as outlined in Lau et al. (2014).

### Sequencing

Extracted DNA from the thermal springs underwent two steps of PCR amplification. DNA was first amplified using universal degenerate primers (27F and 1492R) (DeSantis et al., 2007) and 30 PCR cycles under the conditions outlined in Tekere et al. (2011). After amplification, the entire PCR was loaded onto a 1% agarose gel and an approximately 1500-base pair (bp) band was excised and DNA was recovered using the GeneJET gel extraction kit (Fermentas). The V4-7 region was then amplified using the primers outlined in Table 1 and an annealing temperature of 56°C for 30 cycles. PCR product was then size-selected for 500–600-bp amplicons. Amplicons from each site were pooled at equal concentration and sequenced using a GS-FLX-Titanium (Roche) platform (Inqaba Biotechnology, South Africa).

**Table 1:** **Primers used**.

**Environment**	**Target variable region**	**Annealing temperature**	**Forward (F)/Reverse (R)**	**Primer name**	**Primer sequence (5′ to 3′)**	**Reference for primer**
Thermal Spring	V4-7	56°C	F	530F	GTGCCAGCMGCNGCGG	Dowd et al., 2008
			R	1073R	ACGAGCTGACGACARCCATG	Sundquist et al., 2007
Subsurface	V6	60°C	F	967F	CTAACCGANGAACCTYACC	Sogin et al., 2006
					CNACGCGAAGAACCTTANC	
					CAACGCGMARAACCTTACC	
					ATACGCGARGAACCTTACC	
			R	1064R	CGACRRCCATGCANCACCT	Sogin et al., 2006

The subsurface DNA samples were shipped to the Marine Biological Laboratory (Woods Hole, MA) for sequencing. The V6 region of bacteria was first amplified for 25 cycles using a domain specific amplification by unfused primers (Table 1) and followed by a nested PCR for 5 cycles and fusion primers under the conditions outlined in Eren et al. (2013b). Platinum Taq Hi-Fidelity Polymerase (Life Technologies, Carlsbad CA) was used during PCR amplification. A 101-bp paired-end run was performed on one lane of an Illumina Hiseq 1000.

### Sequence analysis

The V4-7 hypervariable region of the 16S rRNA gene for the thermal spring sites were classified using the Ribosomal Database Project Classifier (Wang et al., 2007). A minimum bootstrap value of 60% at the phylum level was applied to remove sequences with poor annotation quality.

Upon the completion of subsurface V6 sequencing, paired ends were joined and filtered under the requirement that the forward and reverse paired-end reads needed to exhibit 100% consensus in the overlap of the assembled sequence (Eren et al., 2013b). Sequences that matched contaminating sequences previously identified in HiSeq runs at the Marine Biological Laboratory were also removed (Hilary Morrison, personal communication) (Supplement Table 2). Unique sequences and their respective abundance were then identified in the filtered dataset using the USEARCH (Edgar, 2010) “derep_prefix” command. Twelve chimeras were identified and removed using USEARCH's reference based chimera checker (Edgar et al., 2011) and Visualization and Analysis of Microbial Population Structures (VAMPS) refV6 database (http://vamps.mbl.edu/data_downloads/refv6.tgz). This was followed by a *de novo* chimera check (-uchime_denovo) that removed one sequence from the total dataset.

The remaining sequences were annotated using the global alignment for sequence taxonomy (GAST) algorithm (Huse et al., 2008). GAST alignment was performed on a modified version of the refV6 database provided by VAMPS designed to include the V6 regions of bacteria belonging to *Ignavibacteria* (phylum Chlorobi) and South African subsurface clones that have been identified in previous subsurface studies (Takai et al., 2001; Moser et al., 2003, 2005; Kieft et al., 2005; Gihring et al., 2006; Lin et al., 2006a,b; Borgonie et al., 2011; Chehoud, 2011). Rather than providing an *e*-value like BLAST, GAST returns a GAST distance that corresponds to a percent divergence of a queried sequence away from its best match. Huse et al. (2008) reported that a maximum distance cutoff of 0.15 is appropriate to maintain accuracy in taxonomic annotation and, thus, a 0.15 distance cutoff was applied. In order to assess whether or not the fracture had experienced significant contamination from mining processes, we built a database of 16S rRNA sequences that had been previously identified in mine water and mine air of the South African subsurface (Takai et al., 2001; Onstott et al., 2003; Gihring et al., 2006; Lin et al., 2006a,b; Davidson et al., 2011). In total, these studies identified 206 unique sequences of potential mining contaminants from the environment that represented a total of 70 genera. These “potentially contaminating” genera were searched for in the subsurface GAST annotations and their relative abundance was calculated.

To generate operational taxonomic units (OTUs), thermal spring sequences were first aligned using the RDP Infernal Aligner (Version 1.1.rc4) and filtered to include only sequences that aligned within the V4-7 region of the bacterial 16S rRNA gene sequence template (Cole et al., 2014). Subsurface sequences were aligned in mothur (Schloss et al., 2009) against the aligned greengenes “core set” (DeSantis et al., 2006) using a gap penalty of −5. After alignment, a preliminary, pseudo-single linkage clustering step (Huse et al., 2010) was applied to the aligned subsurface sequences using the “pre.cluster” command in mothur (Schloss et al., 2009). The mothur platform was then used to generate a distance matrix (“dist.seqs,” calc=eachgap, countend=F) from which average linkage clustering (“cluster.split”) was performed. OTU clustering at the traditional 0.03 distance (97% of identity or OTU_0.03_) threshold exhibited a high percentage of singletons (>50%) that dramatically inflated the number of observed OTUs in the subsurface sequences (Supplement Table 3). As the total subsurface dataset was very large, shared OTUs were identified between sites through the following procedure: (1) each OTU_0.03_ was represented by the most abundant member; (2) sequences were merged and dereplicated using USEARCH; (3) unique sequences were aligned using the RDP Infernal Aligner (Version 1.1.rc4); (4) a distance matrix was constructed and clustered in mothur as previously described; (5) shared OTUs were identified as those OTUs that clustered at a distance of 0.0049 in mothur.

Singletons were included in this study as the taxonomic distribution of the subsurface dataset was unaffected by singletons at the phylum level (Supplement Figure 1A). The distribution of annotation quality score, given by the GAST distance, for V6 singletons was slightly skewed to the right when compared to the total V6 dataset due to an increased proportion of 0.01–0.02 range GAST distances (Supplement Figure 2). Diversity results after the removal of singletons are reported in Supplement Table 4.

Rarefaction curves, Pielou's Evenness (Equation 1) (Pielou, 1967), Chao1 estimates (Equation 2) (Chao, 1984), and Sørensen (Equation 3) (Sorensen, 1948) and Morisita (Equation 4) (Morisita, 1959) similarity indices for all samples were calculated on the genus level using the Vegan Package (Oksanen et al., 2013) in *R* and following equations:

(1) $$\text{Pielou's Evenness}=\text{H'}/{\text{log}}_{2}({\text{G}}_{\text{obs}})$$

where $H^{\prime }=-\sum_{i\;=\;1}^{G}p_{i}{\text{log}}_{2}p_{i}$, *p*_*i*_ is the proportion of genera *i* in a sample, and *G*_*obs*_ is the number of genera observed in a sample.

(2) $$\text{Chao1}={\text{G}}_{obs}+F_{1}^{2}/2F_{2}$$

where *G*_*obs*_ is the number of genera observed, *F*_1_ is the number of observed singleton genera for a sample and *F*_2_ is the number of observed doubleton genera for a sample.

(3) $$Sørenson\;index=\;\frac{2c}{a+b}$$

where *a* is the number of genera in sample 1, *b* is the number of genera in sample 2, and *c* is the number of genera in common between 1 and 2.

(4) $$\text{Morisita Index}=C_{\lambda }=\frac{2\sum_{i\;=\;1}^{G}n_{1i}n_{2i}}{(\lambda_{1}+\lambda_{2})N_{1}N_{2}}$$

where $\lambda_{j}=\frac{\sum_{i\;=\;1}^{G}n_{ji}(n_{ji}-1)}{N_{j}(N_{j}-1)}$, *n*_*ji*_ is the number of individuals of genera *i* in sample *j* and *N*_*j*_ is the number of individuals in sample *j*.

The Morisita similarity index was calculated to compare sites because, unlike the Sørensen index, it is robust to differences in sample size (Wolda, 1981). The Morisita similarity index was then transformed into what we designate as the “Morisita dissimilarity index” (1-Morisita Index) for subsequent hierarchical clustering.

Although the majority of comparisons in this study were made at the genus level, we also sought to identify what sequences were shared between the thermal spring subsurface sites. To perform this analysis, Bowtie (Langmead et al., 2009) was used to map subsurface V6 sequences to the thermal spring V4-7 dataset under two schemes: (1) a perfect match scenario (-v 0); and (2) a 2 mismatches (-v 2) scenario.

In order to better assess the veracity of the taxonomic inferences drawn from our V6 dataset, we employed oligotyping techniques developed by Eren et al. (2013a) (http://oligotyping.org/) to identify nucleotide level variation within the OTU and genus levels. During oligotyping, the Shannon entropy of each nucleotide position within a group of highly similar sequences, such as an OTU or genus, was calculated and followed by a supervised strategy to identify and decompose the variable sites into oligotypes. For this study, all subsurface sequences found to be closely related (maximum GAST distance of 0.03) to *Candidatus (Ca.) Desulforudis* (*n* = 1381) and *Dehalogenimonas* (*n* = 1,226) were analyzed using this oligotyping pipeline. Shannon entropy profiles were generated using the “entropy-analysis” command on the following groups of subsurface sequences: (1) all sequences related to *Ca. Desulforudis;* (2) all sequences related to *Dehalogenimonas;* and (3) the largest OTU_0.03_ of *Dehalogenimonas* (*n* = 260). These entropy profiles were then used to select the appropriate number of components (−c) for the “oligotype” command. A minimum substantive abundance (−M) of 20 was applied to remove erroneous reads. Notably, *Ca. Desulforudis* and *Dehalogenimonas* were selected for oligotyping because they were the most abundant genera identified within all subsurface sites.

### Statistical analysis

Individual sequences of both subsurface and thermal spring sites were used in the implementation of weighted and unweighted UniFrac significance tests (1,000 iterations, subsample = T) (Lozupone and Knight, 2005). These tests were performed in mothur to determine whether or not the communities within each environment type (thermal spring or subsurface) were significantly different from one another. FastTree (Price et al., 2009) was used to generate the tree for UniFrac significance tests under default settings with *Methanobrevibacter woesei* (NCBI accession number DQ445721) as the outgroup.

A thermal spring geographic distance matrix and (separate) subsurface geographic distance matrix were calculated from the longitude and latitude coordinates of thermal spring and subsurface sampling locations using the “rdist.earth” command in R. Each distance matrix was then correlated to its respective Morisita dissimilarity matrix using the “Pearson” function in Excel. The resulting Pearson product-moment correlation coefficient (PCC) was converted to an *R*^2^ value for future comparisons.

In order to determine the subset of environmental variables with the highest correlation to the Morisita community dissimilarity matrix, the “bioenv” command in the vegan R package was applied (Oksanen et al., 2013). This function builds a Euclidean distance matrix (site × site) based on a subset of scaled environmental variables. The PCC between the environmental distance matrix and phylum-based Morisita community dissimilarity matrix was then calculated. This procedure was performed on environmental distance matrices generated from all possible subsets of environmental variables that the user inputs. For example, environmental distance matrices were generated from all possible combinations of surface elevation, temperature, depth, pH, TDS, Na^+^, K^+^, Ca^2+^, Mg^2+^ of the thermal spring dataset. The correlation between the 511 thermal spring environmental distance matrices and the thermal spring Morisita dissimilarity matrix were then, individually, calculated. The subset of environmental variables that provided the distance matrix with the highest correlation to the Morisita dissimilarity matrix was then returned with a PCC and *R*^2^ value. The same procedure was carried out on the subsurface dataset using the environmental parameters of surface elevation, temperature, depth, pH, TDS, biomass and the concentrations of Na^+^, K^+^, Ca^2+^, Mg^2+^, O_2_, NO^−^_3_, Cl^−^, SO^2−^_4_, dissolved inorganic carbon (DIC), CH_4_, H_2_, and dissolved organic carbon (DOC). Thermal spring and subsurface datasets were analyzed separately due to the fact that the experimental design used to study these two environments was different.

Additionally, pairwise (column × column) Pearson correlations and significance were computed for a matrix containing environmental data and the relative abundance of each phylum (columns) for each sample (rows) using the rcor.test function (ltm R package; Rizopoulos, 2006). This analysis was performed on the thermal spring and subsurface datasets, separately. Notably, the relative abundance of phyla for each group (either thermal spring or subsurface) was calculated from the average of subsampling each sample to 85% of the smallest sample in the group over 1,000 iterations. Significance values (*p*-values) returned by rcor.test were adjusted for multiple tests using the “qvalue” package in R (Dabney et al., 2010). Only pairwise correlations with a *p*-value of <0.05 and *q* of <0.05 were declared significant.

## Results

### Geographic location and geochemistry

All six thermal springs are located within the Limpopo Province of South Africa, within 120 km of each other. Four of the springs, Mphephu, Sagole, Siloam, and Tshipise are located in sediments and volcanic units of the Karoo Supergroup, ranged in elevation from 446 to 841 masl. (Table 2A), and are likely fed by meteoric recharge in the Soutpansberg Mountains formed by Karoo Supergroup sandstones. Eiland and Souting occur in Archean gneiss. They occur in the Lowveld, east of the escarpment that marks the boundary with the Highveld and range in elevation from 389 to 433 masl. (Olivier et al., 2011).

**Table 2A:** **Physical and chemical properties of hot spring water samples^a^**.

**Parameter**	**Eiland**	**Mphephu**	**Sagole**	**Siloam**	**Souting**	**Tshipise**
Latitude (S)	23°39′31″	22°54′20″	22°31′45.4″	22°53′22.6″	23°25′9 ″	22°36′31.5″
Longitude (E)	30°40′23.6″	30°10′38″	30°40′50″	30°11′39″	30°54′44″	30°10′16.7″
Elevation (masl.)	433	796	446	841	389	542
Temperature (°C)	40–42	43	45	63–67	40.1–43.9	58
Estimated depth (m)^b^	700–1050	767–1150	830–1250	1500–2250	730–1100	1267–1900
pH	7.63	8.08–8.19	9.24–9.70	8.8–9.5	7.8	8.3–8.94
TDS (ppm)	>1862	275	237	252	10130	523
O_2_ (M)	n.a.	1.3 × 10^−4^	1.9 × 10^−5^	6.9 × 10^−5^	n.a.	6.1 × 10^−5^
Na^+^ (M)	2.7 × 10^−2^	1.9 × 10^−3^	2.8 × 10^−3^	2.9 × 10^−3^	1.5 × 10^−1^	6.8 × 10^−3^
K^+^ (M)	5.6 × 10^−4^	2.9 × 10^−5^	2.8 × 10^−5^	7.2 × 10^−5^	7.9 × 10^−4^	1.1 × 10^−4^
Ca^2+^ (M)	1.3 × 10^−3^	3.4 × 10^−4^	3.3 × 10^−5^	3.4 × 10^−5^	6.0 × 10^−3^	1.4 × 10^−4^
Mg^2+^ (M)	3.9 × 10^−4^	4.6 × 10^−4^	2.9 × 10^−6^	5.5 × 10^−4^	3.3 × 10^−3^	6.7 × 10^−6^
NO^−^_3_ (M)	4.0 × 10^−5^	3.4 × 10^−5^	<d.l.	<d.l.	4.3 × 10^−5^	9.8 × 10^−6^
Cl^−^ (M)	2.8 × 10^−2^	1.1 × 10^−3^	1.4 × 10^−3^	1.3 × 10^−3^	1.6 × 10^−1^	4.8 × 10^−3^
SO^2−^_4_ (M)	1.5 × 10^−3^	9.5 × 10^−5^	1.9 × 10^−4^	1.1 × 10^−5^	7.9 × 10^−3^	5.5 × 10^−4^
HPO^2−^_4_ (M)	2.6 × 10^−4^	<d.l.	<d.l.	<d.l.	<2.5 × 10^−5^	2.8 × 10^−5^
DIC (M)	<d.l.	2.5 × 10^−3^	2.0 × 10^−3^	2.0 × 10^−3^	n.a.	2.2 × 10^−3^

a This table is adapted from Olivier et al. (2011). Adaptions include the conversion of the various geochemical quantities of Mphephu, Sagole, Siloam, and Tshipise into molar concentrations and the addition of Eiland and Souting into the dataset.

b Estimated depth = (temperature − mean annual surface temperature)/geothermal gradient, where the mean annual surface temperature was assumed to equal 20 °C and the geothermal gradient was assumed to be 20 °C km^−1^ and 30 °C km^−1^.

masl., meters above sea level; n.a., data not available; <d.l., below detection limit.

The six mines are distributed over 500 km of the Kaapvaal Craton and, with the exception of NO14, occur in the Highveld with elevations ranging from 1375 to 1681 masl. (Table 2B). NO14 was collected at the Zondereinde platinum mine, which occurs in the Bushveld Igneous Complex. Geographically, it lies closer to the thermal springs than to Finsch diamond mine (FI88), Beatrix (BE326), or Masimong gold mines (MM51940) (Supplement Figure 3).

**Table 2B:** **Physical and chemical properties of fracture water samples**.

**Parameter**	**Be326_2011**	**Be326_2012**	**Dr5IPC**	**FI88**	**MM51940**	**NO14**	**TT109_Bh2**
Sampling Code	FW250111Bh2	FW270712Bh2	FW280711	FW031012	FW200712	FW130912	FW060312
Latitude (S)	28°14′24.3″	28°14′24.3″	26°25′12″	28°22′43″	27°58′24″	24°49′43″	26°24′55″
Longitude (E)	26°47′49.2″	26°47′49.2″	27°30′10″	23°26′45″	26°52′39″	27°20′26″	27°27′45″
Surface Elevation (masl.)	1375	1375	1654	1545	1386	985	1681
Depth (mbs.)	1339	1339	1046	1056	1900	2100	3136
T (°C)	36.9	38.1	26.8	28.6	40.7	>65^**^	48.7
pH	8.83	8.55	7.39	7.9	7.71–8.18	8.48	8.19
TDS (ppm)	4473	3586	188	1282	3120	2374	296
O_2_ (M)	<3.1 × 10^−7^	9.4 × 10^−6^	1.9 × 10^−6^	1.3 × 10^−6^	1.9 × 10^−6^	3.1 × 10^−6^	6.3 × 10^−6^
CH_4_ (M)	2.0 × 10^−3^	9.0 × 10^−4^	2.6 × 10^−5^	<10^−4^	8.9 × 10^−3^	3.2 × 10^−4^	2.3 × 10^−3^
H_2_ (M)	1.3 × 10^−7^	8.9 × 10^−9^	3.2 × 10^−9^	<10^−8^	1.9 × 10^−7^	4.5 × 10^−6^	3.6 × 10^−7^
Na^+^ (M)	7.8 × 10^−2^	4.8 × 10^−2^	2.2 × 10^−3^	9.9 × 10^−3^	4.5 × 10^−2^	9.4 × 10^−2^	3.4 × 10^−3^
K^+^ (M)	7.3 × 10^−4^	8.5 × 10^−4^	5.6 × 10^−5^	1.8 × 10^−4^	4.0 × 10^−4^	4.1 × 10^−3^	4.2 × 10^−5^
Ca^2+^ (M)	2.9 × 10^−3^	3.9 × 10^−3^	5.0 × 10^−4^	5.6 × 10^−3^	1.9 × 10^−3^	2.5 × 10^−3^	5.2 × 10^−4^
Mg^2+^ (M)	5.6 × 10^−5^	2.6 × 10^−5^	4.0 × 10^−4^	1.5 × 10^−4^	9.9 × 10^−5^	6.4 × 10^−4^	3.4 × 10^−5^
NO^−^_3_ (M)	3.7 × 10^−7^	6.0 × 10^−6^	1.5 × 10^−6^	1.5 × 10^−5^	9.7 × 10^−7^	n.a.	1.1 × 10^−7^
Cl^−^ (M)	7.0 × 10^−2^	6.2 × 10^−2^	1.4 × 10^−3^	2.2 × 10^−2^	5.5 × 10^−2^	5 × 10^−2^	3.2 × 10^−3^
SO^2−^_4_ (M)	1.4 × 10^−4^	6.2 × 10^−4^	1.4 × 10^−4^	3.0 × 10^−4^	7.0 × 10^−6^	n.a.	1.0 × 10^−4^
HPO^2−^_4_ (M)	<1.1 × 10^−6^	3.0 × 10^−7^	1.6 × 10^−7^	3.5 × 10^−7^	<3.2 × 10^−7^	1.1 × 10^−7^	1.2 × 10^−6^
DOC (M)	1.5 × 10^−5^	2.9 × 10^−5^	8.5 × 10^−5^	1.3 × 10^−4^	4.5 × 10^−5^	2.6 × 10^−5^	3.9 × 10^−5^
DIC (M)	5.1 × 10^−4^	3.3 × 10^−4^	2.4 × 10^−3^	5.4 × 10^−5^	4.3 × 10^−4^	5.2 × 10^−4^	6.9 × 10^−4^
DIC Age (kyr)	>32	>20	16–23	>32	>32	4.28	16-21

** 65°C was the upper temperature limit of the thermometer used.

masl., meters above sea level; mbs., meters below surface; n.a., data not available.

Siloam was the warmest thermal spring with measured temperatures of 63–67°C, comparable to the hottest subsurface site, NO14, with a measured temperature above 65°C. The shallowest subsurface sites (Dr5IPC and FI88) contained the coolest water sampled (26.8°C and 28.6°C, respectively) and were more than 10°C cooler than the coolest thermal spring sampled, Eiland (40–42°C). The thermal spring temperatures were consistent with a minimum depth of ground water circulation ranging from 700–2250 m (Table 2A). These minimum depth estimates overlapped the 1046–3136 m below surface (mbs.) sampling depths of the fracture water samples.

**Figure 1:**
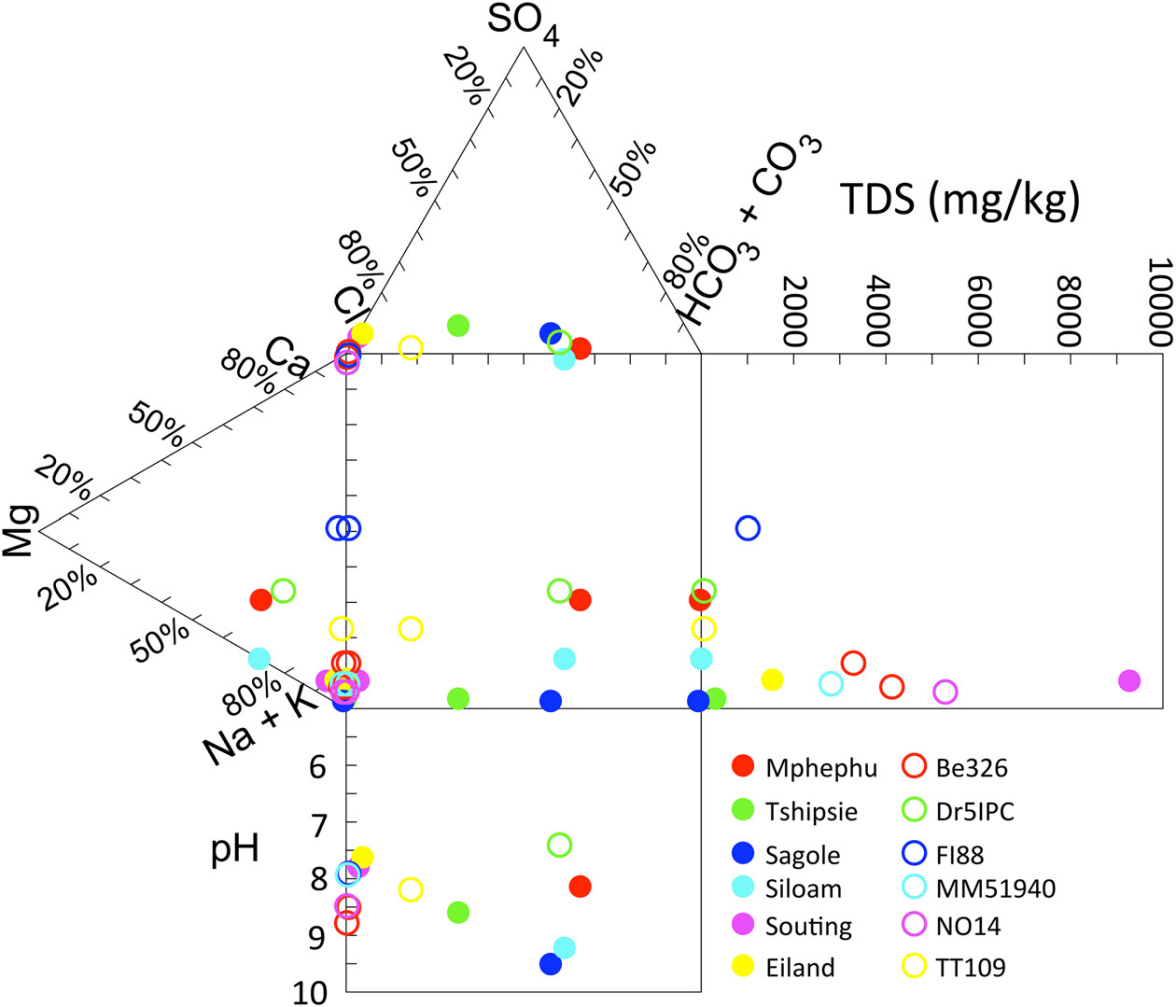
**Durov plot of thermal spring and subsurface chemistry**. Geochemical parameters of thermal spring (filled circles) and subsurface (open circles) are plotted on a Durov Plot.

The pH of the six thermal springs sampled ranged from 7.6 (Eiland) to 9.7 (Sagole), whereas the pH of the subsurface fracture water was more narrowly restrained from 7.4 (Dr5IPC) to 8.3 (Be326). The TDS of the thermal springs ranged from fresh to brackish (237 ppm for Sagole to 10,130 ppm for Souting), overlapping the TDS of the fracture water, which ranged from 188 (Dr5IPC) to 4473 ppm (Be326_11). The O_2_ concentration of the thermal springs ranged from 20 μM (Sagole) to 130 μM (Mphephu) and were elevated above the subsurface O_2_ concentrations that ranged from below detection (<1 μM) to 9.4 μM (Be326_2012). The thermal springs located in the Karoo Supergroup had a sodium carbonate composition, whereas the thermal springs located in the Archean gneiss had a sodium chloride composition (Olivier et al., 2011). The fracture water composition ranged from a calcium, magnesium carbonate (Dr5IPC) to sodium chloride composition (Be326). When plotted on a Durov projection Dr5IPC was geochemically very similar to the Mphephu thermal spring water (Figure 1).

### Sequencing summary

A total of 3,055 sequences from the V4-7 hypervariable region of the 16S rRNA gene were generated for the thermal spring sites. Sequences generated for each site varied in length (average length 360 ± 144 bp) (Supplement Figure 4) and number (Table 3). A total of 291 genera within 17 phyla were observed in the thermal springs after annotation quality filtering was applied.

**Table 3:** **Summary of diversity statistics for thermal spring and subsurface samples**.

**Site**	**Number of reads**	Number of reads classified by RDP* or GAST^^^	**Number of genera observed**	**Pielou's evenness**	**Chao1^a^**
Eiland	720	698 (97%)^b^	49 (62%)^c^	0.57	79 ± 19
Mphephu	721	673 (93%)^b^	92 (68%)^c^	0.69	135 ± 20
Sagole	794	662 (83%)^b^	113 (59%)^c^	0.72	190 ± 30
Siloam	568	515 (91%)^b^	119 (57%)^c^	0.78	209 ± 34
Souting	132	120 (91%)^b^	39 (57%)^c^	0.83	68 ± 21
Tshipise	120	110 (92%)^b^	30 (47%)^c^	0.74	64 ± 31
Total thermal springs	3,055	2,778 (91%)^b^	291 (70%)^c^	0.73	412 ± 31
Beatrix Au Mine—Be326_2011	14,227	13,328 (94%)^d^	551 (77%)^c^	0.80	716 ± 37
Beatrix Au Mine—BE326_2012	23,343	21,806 (93%)^d^	634 (86%)^c^	0.80	739 ± 25
Driefontein Au Mine—Dr5IPC	18,215	15,876 (87%)^d^	549 (84%)^c^	0.80	653 ± 24
Finsch Diamond Mine—FI88	18,719	17,282 (92%)^d^	573 (79%)^c^	0.78	725 ± 34
Masimong Au Mine—MM51940	22,984	20,768 (90%)^d^	655 (86%)^c^	0.80	760 ± 24
Zondereinde Pt Mine—NO14	22,237	20,676 (93%)^d^	640 (86%)^c^	0.79	744 ± 24
Tau Tona Au Mine—TT109	19,367	17,792 (92%)^d^	569 (83%)^c^	0.77	689 ± 27
Total subsurface	139,092	127,528 (92%)^d^	874 (88%)^c^	0.77	991 ± 28

a Chao1 is estimated at the genus level.

b Minimum bootstrap value of 60% at phylum level.

c Observed Genera/Chao1 in percent.

d Maximum distance of 0.15.

The number of V6 sequences passing the 100% overlap quality filtering was 139,092 and ranged from 14,227 (Be326_2011) to 23,343 (Be326_2012) sequences per site. After annotation using GAST, 87% (Dr5IPC) to 94% (Be326_2011) of the sequences were assigned an annotation and used in downstream analyses (Table 3). The number of genera identified for each site ranged from 549 (Dr5IPC) to 655 (MM51940), and the number of OTU_0.03_ ranged from 5,952 (Be326_2011) to 9,456 (MM51940). A total of 874 genera spanning 44 phyla were observed in the subsurface sequences. When singletons were removed, a total of 616 genera were identified and the number of OTU_0.03_ ranged from 2,478 (Be326_2011) to 3,987 (Be326_2012) (Supplement Table 3). Of the 258 genera that were represented solely by a single sequence at any subsurface site, 125 were “true” singletons—meaning that the genus was represented by only 1 sequence in the combined subsurface dataset. Upon removal of the 258 singleton genera, the taxonomic distribution and similarity indices remained largely the same (Supplement Figure 1) and, thus, singletons were included in the subsequent analyses.

### Taxonomic distribution of thermal springs

**Figure 2:**
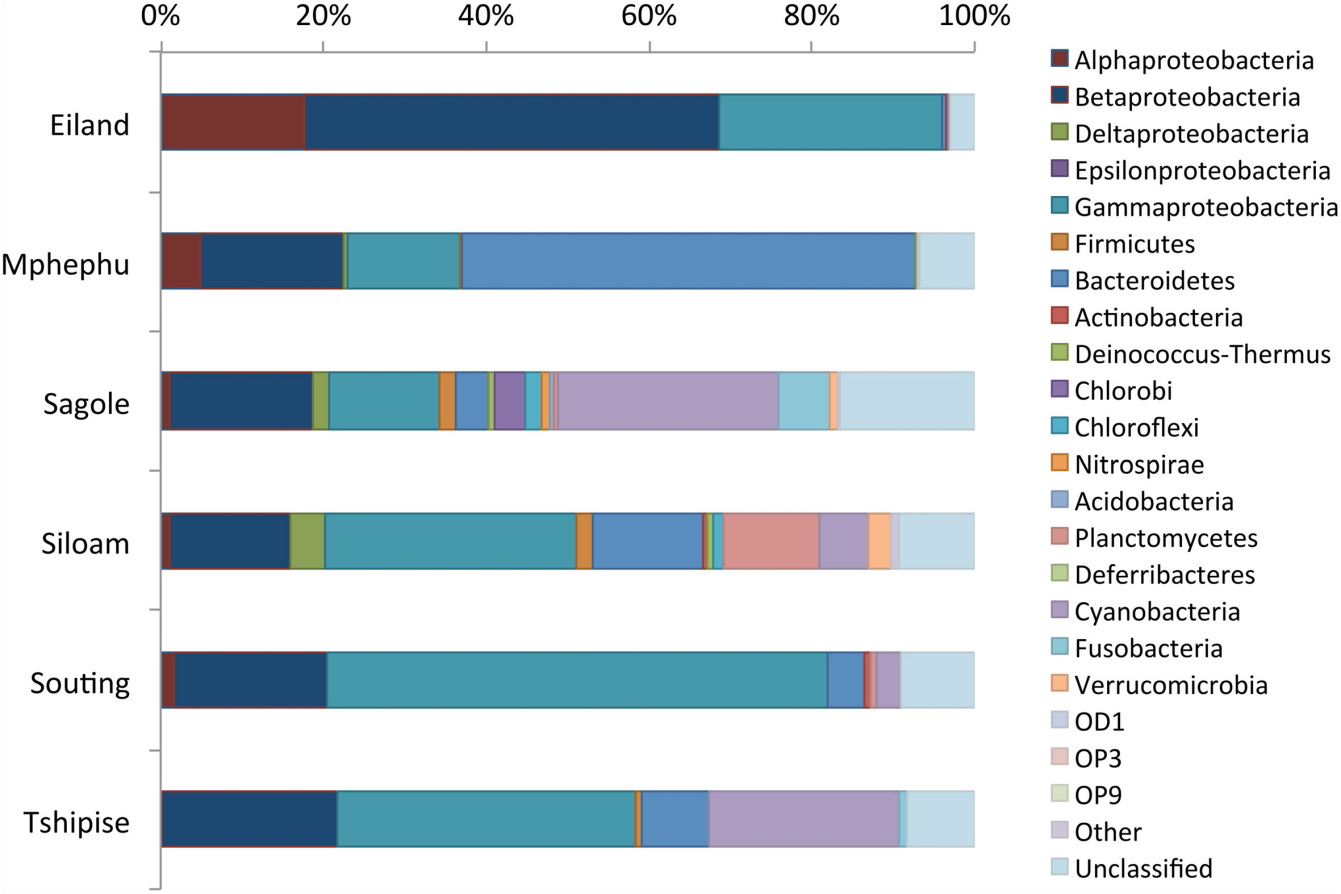
**Thermal spring taxonomic distribution**. A bar plot of the relative abundance (x-axis) of various phyla (color) per thermal spring site (y-axis). Due to its high relative abundance, the phylum proteobacteria was split into its corresponding classes. Members of the “Other” bin include: Armatimonadetes, Spirochaetes, SR1, and Synergisetes.

Rarefaction curves (Supplement Figure 5) revealed that sequences from Eiland, Mphephu, Sagole and Siloam thermal springs captured more of the diversity within those sites than the limited number of sequences from Souting (*n* = 132) and Tshipise (*n* = 120) thermal springs did. A comparison of the observed number of genera vs. the number estimated by the Chao1 parameter (Chao, 1984) suggested that the sequences represent 47% (Tshipise) to 68% (Mphephu) of the predicted total number of genera present in the thermal springs (Table 3). All thermal spring samples yielded taxonomic distributions that were found to be significantly different (*p* < 0.001) from one another through both weighted and unweighted pairwise Unifrac significance tests (Lozupone and Knight, 2005). Four of the six thermal springs (Eiland, Siloam, Souting, and Tshipise) were found to be dominated (>50%) by Proteobacteria, whereas approximately 55% of Mphephu's sequences belonged to Bacteroidetes. Sagole's most abundant phylum was Cyanobacteria (32%) (Figure 2). Souting was found to be the thermal spring site with the highest evenness (Pielou's evenness = 0.83; Table 3) with 30 different genera of Gammaproteobacteria present. Eiland exhibited the lowest Pielou's evenness (0.57) with 60% of sequences belonging to two genera of Proteobacteria: *Hydrogenophaga* (class Betaproteobacteria) and *Stenotrophomonas* (class Gammaproteobacteria). Sequences from Siloam contained the highest number of unique genera (119), whereas Tshipise contained the fewest unique genera (30) (Table 3). No sequences derived from Tshipise were related to Alphaproteobacteria.

### Taxonomic distribution of south african subsurface sites

**Figure 3:**
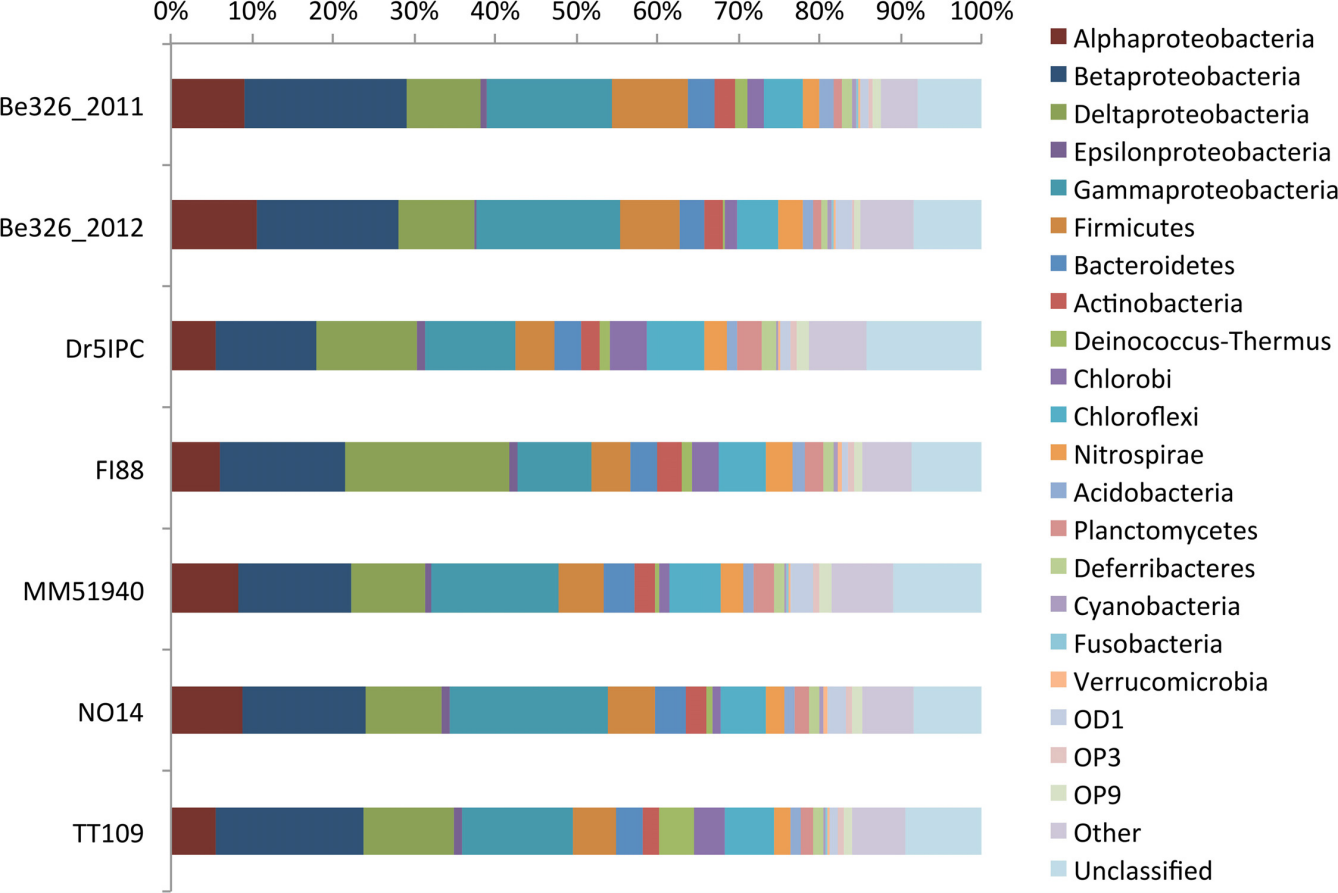
**Subsurface taxonomic distribution**. A bar plot of the relative abundance (x-axis) of various phyla (color) per subsurface site (y-axis). Due to its high relative abundance, the phylum proteobacteria was split into its corresponding classes. Members of the “Other” bin include: BRC1, Caldiserica, Chlamydiae, Chloroplast, Crenarchaeota, Dictyoglomi, Elusimicrobia, Euryarchaeota, Fibrobacteres, Gemmatimonadetes, Lentisphaerae, Mitochondria, OP1, OP2, OP8, OP10, OP11, Spirochaetes, Synergistetes, TA06, Tenericutes, TG-1, Thermotogae, TM6, TM7, WS1, WS3, WS6, and Zetaproteobacteria.

Although the subsurface rarefaction curves (Supplement Figure 5) did not attain a plateau, the reduced slope of each curve toward the terminus suggested that the sequences for each of these samples are representative of their bacterial community. Across all sites, Proteobacteria were the most abundant phylum, with relative abundances ranging from 49% (Dr5IPC) to 59% (Be326_2012) (Figure 3). Weighted, pairwise Unifrac significance tests indicated that all sites were significantly different (*p* < 0.001); however, unweighted, pairwise UniFrac significance tests revealed that only 7 of the 21 pairs of sites were found to be significantly different (*p* < 0.05) from one another (Supplement Figure 6). All subsurface sites displayed a Pielou's evenness between 0.77 (TT109) and 0.80 (MM51940) (Table 3). Previous studies of South African fracture fluids (Takai et al., 2001; Moser et al., 2003, 2005; Kieft et al., 2005; Gihring et al., 2006; Lin et al., 2006a,b; Borgonie et al., 2011) identified a total of 243 unique genera in the South African subsurface (Chehoud, 2011). Of these 243 genera, 139 were observed in our V6 datasets.

### Potential contaminants in the subsurface

Within the subsurface V6 dataset, 49 of the 70 designated contaminant genera (see Section Sequence Analysis) were identified in our subsurface samples. The relative abundance of these genera ranged from 4.3% (Dr5IPC) to 8.6% (Be326_2012) of each site's V6 dataset. Unfortunately, we cannot unambiguously determine whether or not these genera are true contaminants because mining water is a mixture of water released during dewatering of fractures and water derived from regional water supplies; however, we make the conservative assumption that they could be contaminants that penetrated the fractures during drilling with mining water.

### Oligotyping

**Figure 4:**
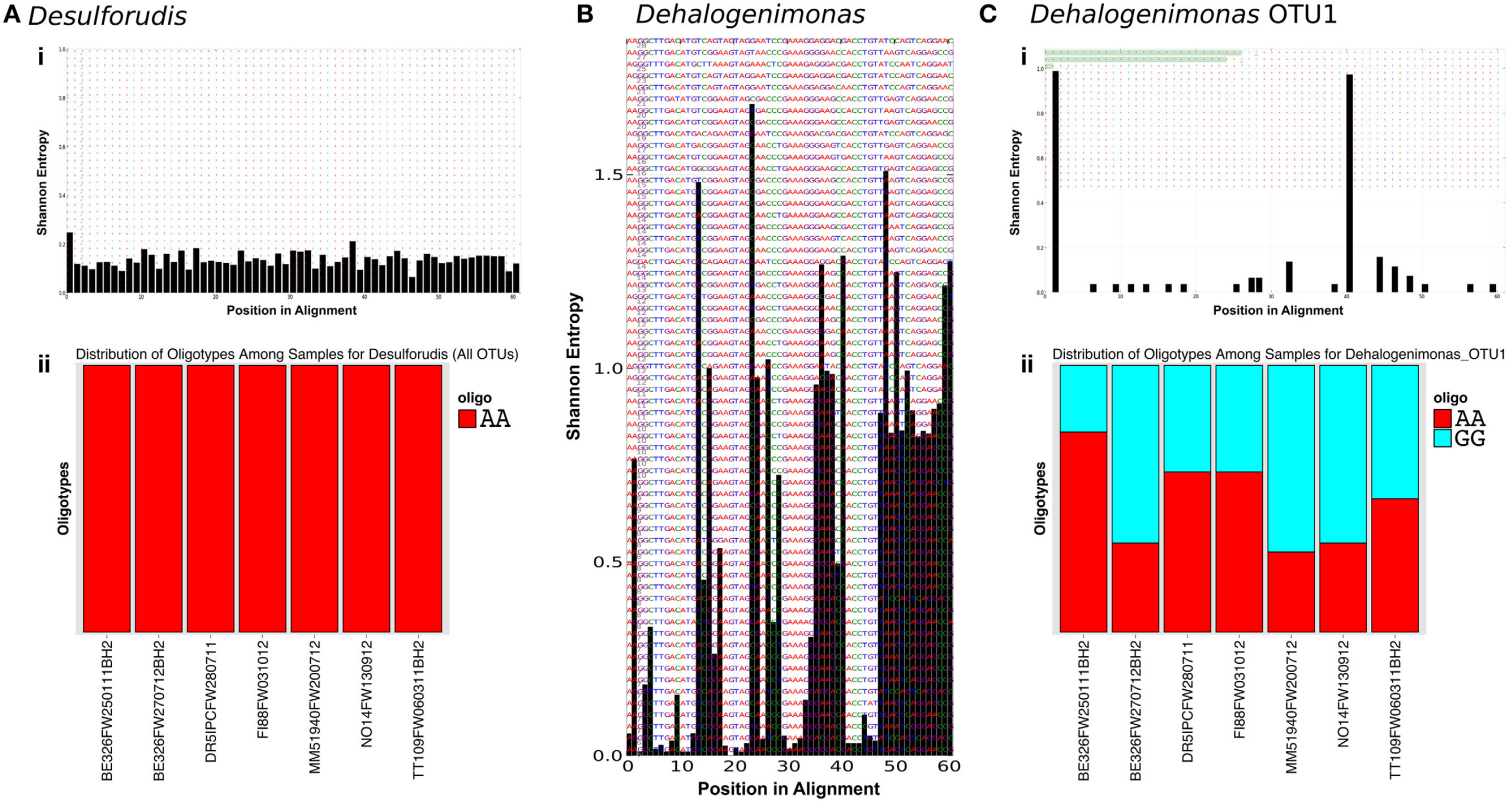
**Oligotypes of abundant genera**. Entropy profiles for *Desulforudis* **(Ai)**, *Dehalogenimonas* **(B)**, and *Dehalogenimonas* OTU1 **(Ci)** are a discrete xy plot where the x-axis is the nucleotide position in the alignment and the y-axis is the calculated Shannon entropy for that position. Figures **(Aii)** and **(Cii)** display the relative abundance of the oligotypes observed. For *Desulforudis*, only one oligotype (AA) was observed while *Dehalogenimonas* revealed two oligotypes (AA, GG). The position of these distinctive nucleotides can be found in the entropy plot of **(Ci)**. The elevated entropy at positions 1 and 40 reveal that these positions are where the single nucleotide changes occur.

Oligotyping was performed on all subsurface sequences related to the two most abundant subsurface genera, *Ca. Desulforudis* and *Dehalogenimonas*, to identify nucleotide level variation within the OTU and genus level. A total of 1,381 sequences were identified as related to *Ca. Desulforudis* while 1,226 sequences were identified as related to *Dehalogenimonas* representing 62 and 164 OTUs at the 97% identity level, respectively. Entropy profiles for all sequences from each genus can be found in Figures 4Ai,B. Eren et al. (2013a) reported that random sequencing errors generate entropy values near or below 0.2. For *Ca. Desulforudis*, 59 of the 61 positions exhibited Shannon entropy values less than 0.2, while the remaining two positions had entropy values of 0.2487 and 0.2125. Oligotyping of these 2 positions (−c 2, −M 20) revealed that only one oligotype was present (Figure 4Aii), suggesting that the *Ca. Desulforudis* genus identified within the subsurface contain highly similar V6 regions. On the other hand, the Shannon entropy profile of all sequences related to *Dehalogenimonas* showed that 33 of the 61 positions of the V6 region contained entropy values greater than 0.2 (Figure 4B). Due to the large number of high entropy positions in *Dehalogenimonas*-related V6 sequences, oligotyping was performed on the most abundant OTU_0.03_ (*n* = 260) instead of the complete set of sequences assigned to this genus. For this OTU, Shannon entropy profiles indicated two high entropy positions that decomposed into 2 oligotypes (−c 2, −M 20; Figure 4C).

### Shared taxa

**Figure 5:**
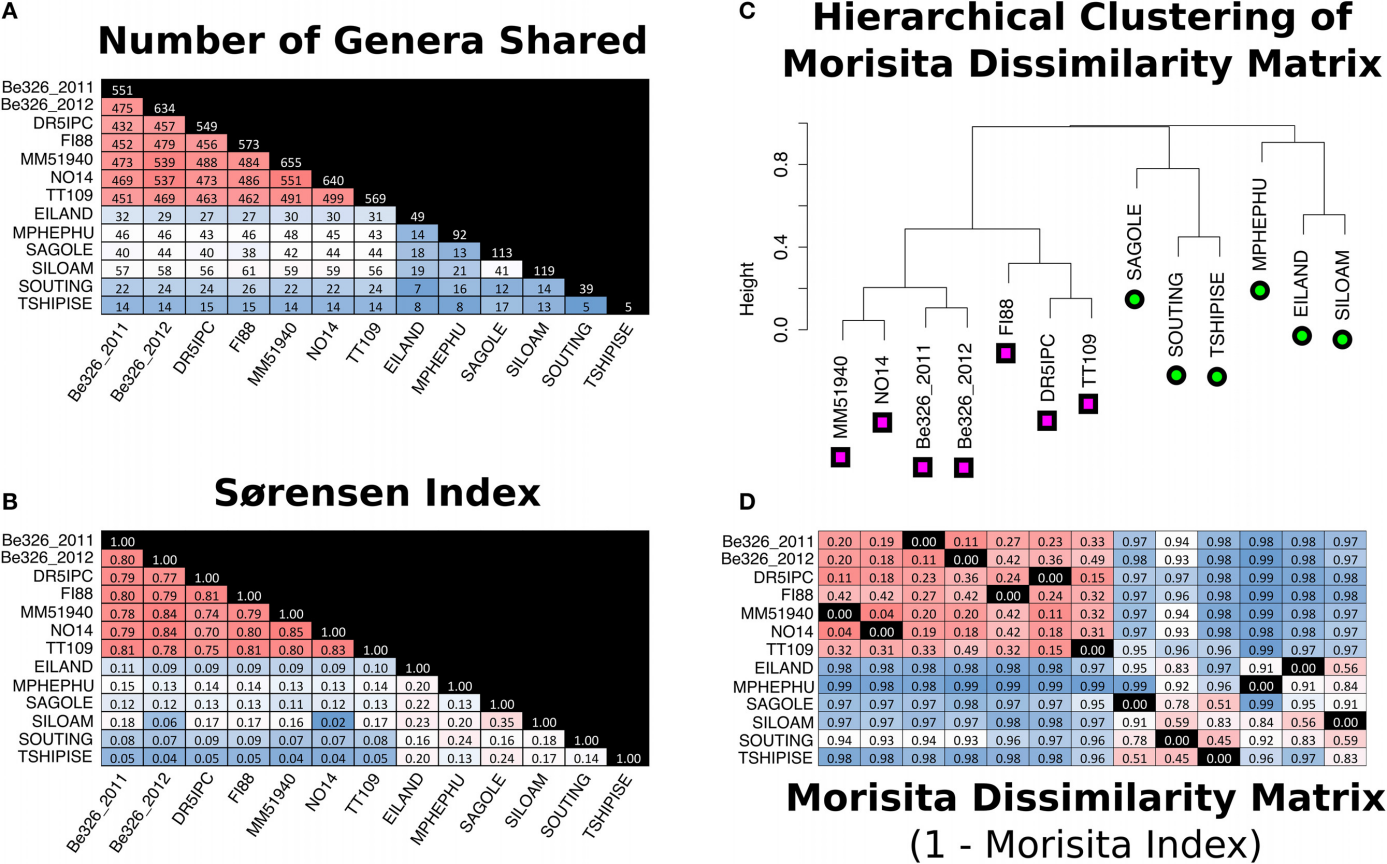
**Measurements of similarity between thermal spring and subsurface sites**. Pair-wise comparisons have been made by counting the total number genera shared between sites **(A)** and calculation of the Sorensen similarity index **(B)** and Morisita dissimilarity indices (1-Morisita index) **(D)**. Color-coding of **(A,B,D)** are purely for visual aid where red indicates more similar pairs and blue is indicative of more distant pairs. The diagonal of **(A)** indicates the number of genera identified in the sample. **(C)** is a visual representation of the hierarchical clustering of the Morisita dissimilarity matrix **(D)**. Subsurface sites in **(C)** are labeled with pink squares and thermal spring sites are labeled with green circles. The columns of **(D)** are indicated by the branches of **(C)** whereas the columns of **(A,B)** are labeled. Black squares labeled 0.00 in **(D)** indicate the same sample in the row and **(C)**'s branch.

There was a very large difference between the communities of individual thermal springs. No individual sequence was found in multiple thermal spring sites, however, when sequences were clustered into OTUs at a distance of 0.03, two OTU_0.03_ were shared between Eiland and Mpephu, one OTU_0.03_ was shared between Eiland and Sagole, and one OTU_0.03_ was shared between Sagole and Siloam. At the genus level, Sagole and Siloam shared the greatest number of genera (*n* = 41; Sørensen index = 0.35) while Souting and Tshipise shared the lowest number of genera (*n* = 5; Sørensen Index = 0.14) (Figures 5A,B).

**Figure 6:**
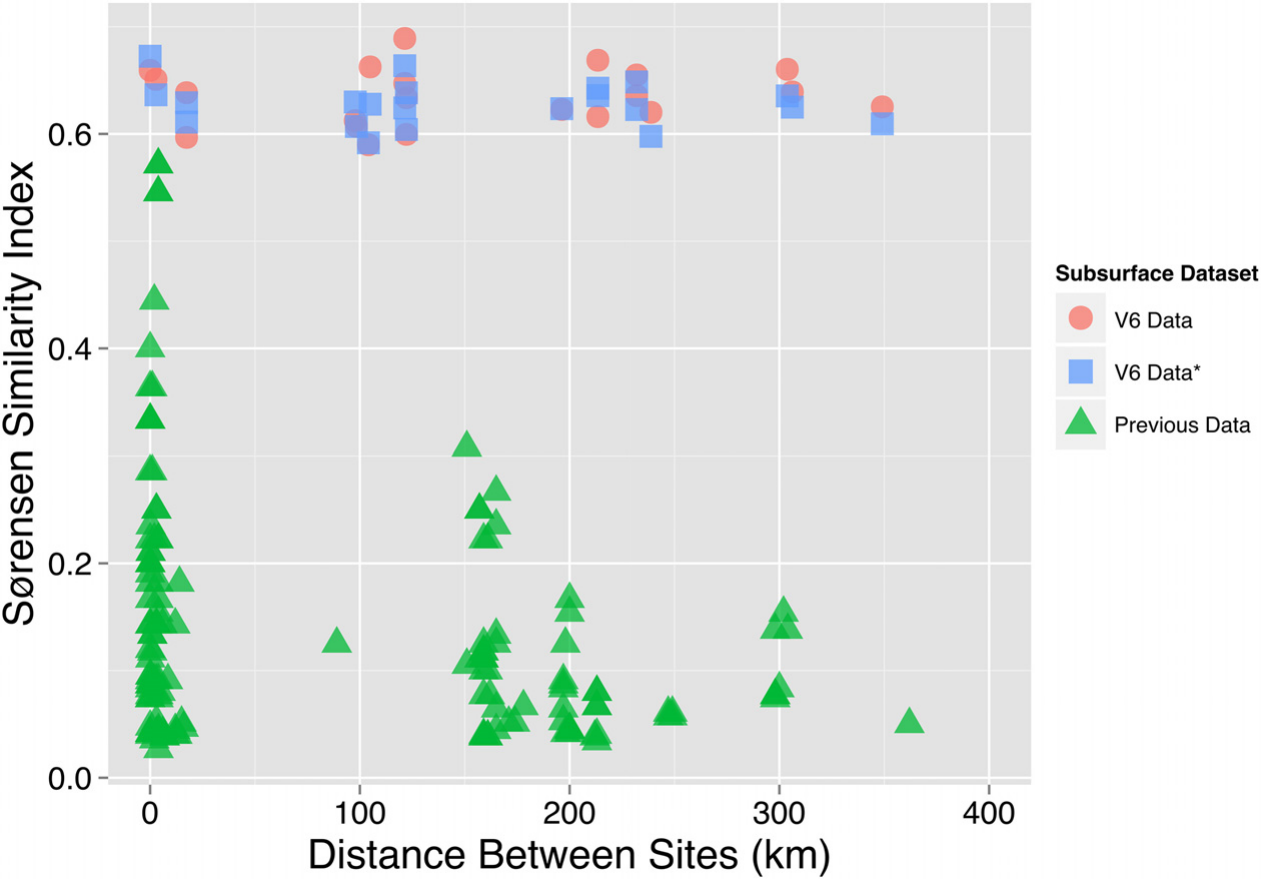
**Sørensen similarity index in relation to geographical distance**. The OTU_0.03_ Sørensen similarity index (y-axis) of subsurface sites is shown in relationship to the geographical distance between the two sites used to calculate the Sørensen index (x-axis). The V6 data used in this study is displayed as both the Sørensen indices of the total subsurface OTU_0.03_ dataset (V6 Data, red circle) and a rarefied OTU_0.03_ Sørensen Index (V6 Data*, blue square). The rarefied OTU_0.03_ Sørensen index was calculated by averaging the result of 100 iterations of subsampling the OTU_0.03_ community data table to 85% of the smallest sample. A third set of OTU_0.03_ Sørensen similarity indices and distances (Previous Data, green triangle) was calculated from previous South African subsurface studies (Takai et al., 2001; Moser et al., 2003, 2005; Kieft et al., 2005; Gihring et al., 2006; Lin et al., 2006a,b; Borgonie et al., 2011; Chehoud, 2011).

All seven subsurface samples shared 220 genera and 1410 V6 sequences. Shared V6 sequences were present at similar relative abundances within each subsurface site (Supplement Figure 7). NO14 and MM51940 shared the most genera (*n* = 551) and exhibited the highest Sørensen indices (0.85) (Figures 5A,B, respectively). At the OTU_0.03_ level, Sørensen similarity indices ranged from 0.59 to 0.69 between sites—a range higher than previously reported in the South African subsurface (Figure 6). When singleton genera were removed, Sørensen indices increased slightly (0.70–0.85 to 0.80–0.88), while Morisita dissimilarity indices remain unchanged (Figures 5B,D; Supplement Figure 1B,D).

A direct alignment of subsurface V6 sequences onto the collection of thermal spring sequences allowed us to determine whether or not subsurface V6 sequences were present in the thermal springs. However, only 367 (12%) of thermal spring sequences were long enough to capture the V6 region of the 16S rRNA gene and, therefore, this direct comparison of V6 sequences is not a complete representation of the presence or absence of V6 sequences shared between both environments. Despite this shortcoming, through alignment, we found that 13 thermal spring V6 sequences were present in the subsurface samples (Supplement Figure 8A). These shared sequences were related to Proteobacteria, Bacteroidetes, Deinococcus-Thermus, and Fusobacteria. Five of the 13 shared sequences were present in all seven subsurface V6 datasets. When a 2-bp mismatch was allowed, only 2 additional thermal spring sequences [relating to *Azonexus* (Proteobacteria) and *Propionigenium* (Fusobacteria)] were identified in the subsurface (Supplement Figure 8B). As the occurrence of sequences within multiple subsurface samples may be a result of contamination from surface water sources during mining, we also compared the genera of sequences shared between the thermal spring and subsurface environments with the genera in our mining contaminant dataset (see Section Sequence Analysis). We found that 4 of the 12 genera (*Blastomonas, Novosphigmodium, Meithermus, and Rheinheimera*) identified in both thermal springs and subsurface sites were also identified as possible mining contaminants.

### Relationship of taxonomy to environment

After performing a pairwise-Pearson correlation for all combinations of phyla and environmental variables in the thermal spring and subsurface datasets (separately), no significant correlations were found. However, we were able to identify which sets of environmental variables had the highest correlation to each environment's respective Morisita dissimilarity matrix. The Morisita dissimilarity matrix of the thermal springs was most correlated to a Euclidian distance matrix constructed from the scaled values of surface elevation and pH (*R*^2^ = 0.20). The subsurface Morisita dissimilarity matrix was most correlated to a Euclidian distance matrix constructed from the scaled values of surface elevation, pH, TDS and the concentrations of O_2_, Ca^+^, DIC, CH_4_, and Cl^−^ (*R*^2^ = 0.83). The geographical distance matrices were not highly correlated to the thermal spring (*R*^2^ = 0.03) or subsurface (*R*^2^ = 0.12) Morisita dissimilarity matrices.

## Discussion

The similarity in geochemistry of the Limpopo thermal springs and South African subsurface suggested that the springs might provide a portal to the deep biosphere. However, although Proteobacteria dominated the majority of thermal spring and subsurface sites, there are more differences than similarities when the microbial communities of the thermal springs and subsurface sites are compared.

### Differences in the diversity of thermal springs and subsurface sites

In order to compare the community composition of various environments, Morisita dissimilarity indices (MDI) were calculated for all pairs of samples (Figure 5D). Hierarchical clustering of this dissimilarity matrix indicates that the communities of thermal springs and subsurface environments are distinct from one another (Figure 5C). Although Mphephu and Dr5IPC were, geochemically, the most similar thermal spring and subsurface fracture, they are, taxonomically, further apart from each other (MDI: 0.99) than Mphephu is from the geochemically quite dissimilar thermal spring of Souting (MDI: 0.92). The number of genera observed in the thermal springs (30–119) was much lower than in the subsurface sites (549–655), although this may reflect the smaller sample volumes and lower number of reads of the thermal spring samples (see Section Comments on Experimental Design). Within environment types, the microbial communities of thermal springs exhibited higher MDIs (MDIs 0.45–0.99) when compared to the subsurface samples (MDIs 0.04–0.49). It has been well reported that the microbial communities of hot springs are highly variable due to effects of water flow, hail, trampling, and seasonal variation (Brock and Brock, 1969; Ferris et al., 1997; Ward et al., 1998; Fouke et al., 2003). Tekere et al. (2012) attributed the distinctive taxonomic distributions of the Limpopo thermal springs to differences in geographical settings such as the large populations of fermentative Bacteroidetes found at the Mphephu springs—a site with a high input of organic carbon from the surrounding vegetation. Furthermore, as the thermal spring dataset is a combination of planktonic and biofilm communities, it is likely that the biofilm communities play a large role in the inter-site variation observed in the thermal springs. Fouke et al. (2003) and Meyer-Dombard et al. (2011) have reported that the communities formed on a biofilm can vary locally even when fed by the same source water. For the thermal springs sampled, only one section of each spring's biofilm was collected and analyzed in conjunction with 2 L of filtered water. The small sample size in the thermal spring dataset undoubtedly amplifies local effects within a thermal spring community and further elevates the amount of variation observed between thermal spring samples.

### Ubiquitous microorganisms

The only genus observed in all 13 datasets was a heterotrophic, non-spore forming Gammaproteobacteria, *Rheinheimera*. The relative abundance of this genus was highly variable between thermal spring sites (maximum: 35% for Tshipise; minimum: 0.1% for Eiland) and only a trace component of subsurface samples (<0.1%). Isolated species of *Rheinheimera* have been found in marine and soil environments with a maximum growth temperature of ~40°C (Brettar et al., 2006; Yoon et al., 2007; Zhang et al., 2008), making it surprising that *Rheinheimera* would dominate a warmer thermal spring site and not the other, cooler locations. This suggests that the strain of *Rheinheimera* observed in Tshipise is adapted to higher temperatures than previously isolated species. Despite the low relative abundance of *Rheinheimera* in the subsurface samples, *Rheinheimera* has been identified in two other South African sites. One was an isolate from a water sample from the Transvaal dolomite chamber of Driefontein 4 shaft, Dr4IPC, (NCBI accession number DQ133435) and the other was from an earlier 16S rRNA gene clone library of Be326 (*n* = 3) (Borgonie et al., 2011), the same borehole that was sampled twice in this study. Due to the low relative abundance of *Rheinheimera*-related sequences in the subsurface V6 datasets and the fact that it is a mesophilic aerobic heterotroph, it is unlikely that *Rheinheimera* plays an important role in subsurface ecology. Instead, *Rheinheimera* is likely a product of either mining contamination or preserved extracellular DNA in the subsurface. *Rheinheimera* has been identified as a potential mining contaminant as it was found in the service water of Evander mine (Davidson et al., 2011). Although no efforts have been made to identify extracellular DNA in the South African subsurface, the preservation of extracellular DNA in marine sediments has been well reported (Danovaro et al., 2005; Coolen and Overmann, 2007; Corinaldesi et al., 2008, 2011) and suggested to occur due to the adsorption of DNase onto sediment particles (Demanèche et al., 2001) as well as the adsorption of extracellular DNA onto mineral and organic particles under anoxic conditions (Coolen and Overmann, 2007). If similar processes are occurring in the terrestrial subsurface fracture water, then it will be difficult to elucidate the authenticity of low abundance genera like *Rheinheimera* based on V6 amplicon studies.

Although not identified in the thermal springs, a member of the shared subsurface community that is worth mentioning is the sulfate reducing bacterium *Ca. Desulforudis.* This firmicute was found within all subsurface sites sampled and appears to be an important member of the subsurface community. In 2008, *Ca. Desulforudis* was identified as the sole member of a subsurface fracture community (Chivian et al., 2008) and, since then, *Ca. Desulforudis* has been identified in other South African subsurface studies (e.g., Davidson et al., 2011). Additionally, close relatives of *Ca. Desulforudis* have also been identified at other subsurface sites (Itävaara et al., 2011; Suzuki et al., 2013; Tiago and Veríssimo, 2013). Interestingly, despite the slight variations in the partial 16S rRNA gene sequences reported for *Ca. Desulforudis* found in geographically dispersed fractures of the South African subsurface (Gihring et al., 2006; Chivian et al., 2008; Davidson et al., 2011), these sequences yield only one oligotype of *Ca. Desulforudis* V6. The variation in 16S rRNA gene of South African *Ca. Desulforudis* was found largely within the V3-5 hypervariable regions, not in the V6. Such conservation of the V6 is uncommon; other bacterial species, such as those relating to *Dehalogenimonas* (Figure 4C), occur as a variety of strains and oligotypes. It is unclear why the V6 region of *Ca. Desulforudis* is well conserved while the V3-5 is not. However, conservation of the *Ca. Desulforudis* genome has been reported previously, as only 32 single nucleotide polymorphisms were identified in the *Ca. Desulforudis* metagenome (Chivian et al., 2008). With such conservation of *Ca. Desulforudis's* 16S rRNA gene in the South African subsurface, it will be interesting to determine how conserved the whole genomes of *Ca. Desulforudis* are across sites in the subsurface of South Africa.

### Increased occurrence of rare genera in the subsurface

In the subsurface, the identification of low abundance genera like *Rheinheimera* has increased the alpha diversity beyond what had been reported previously. A similar increase in alpha diversity with V6 sequencing was reported by Sogin et al. (2006) and was attributed to the successful observation of low-abundance organisms that comprise the “rare biosphere.” However, the authenticity of this “rare biosphere” has been debated as the propagation of sequencing error may have greatly increased the apparent diversity (Reeder and Knight, 2009). In the case of the subsurface samples reported here, sequencing error has already been accounted for through the use of the 100% overlap filter. However, as exhibited by the oligotyping of *Ca. Desulforudis*-related sequences, there is a large amount of noise surrounding “authentic” V6 sequences that is most likely derived from PCR error (Eren et al., 2013b). This PCR noise is so large that the single-linkage preclustering and subsequent average linkage clustering of pairwise aligned sequences (SLP-PWAL) strategy proposed by Huse et al. (2010) did not effectively reduce the number of OTUs. Based on these results, it is conceivable that other artificial sequences may have been generated during PCR and, falsely, increased the alpha diversity of the subsurface sites.

### Dispersion of subsurface populations

Aside from identifying a large number of rare sequences, we also observe over 200 ubiquitous subsurface genera. This degree of overlap in South African subsurface communities has not been previously observed. To illustrate this, we compared the OTU_0.03_ Sørensen similarity index for all pairs of datasets generated in the South African subsurface and compared them with the OTU_0.03_ Sørensen indices of this study (Figure 6). When these Sørensen similarity indices were plotted against the distance between the pair of sites being compared, our V6 dataset maintains a steady Sørensen similarity index (0.59–0.69) across geographical distance while the historical data shows a decay in Sørensen similarity index values with distance (Chehoud, 2011). Accepting that the level of similarity as defined by our OTU_0.03_-based Sørensen similarity indices (0.59–0.69) is not an artifact of methodology, then the lack of an obvious distance-decay relationship across hundreds of kilometers (and kilometers vertically) is challenging to explain.

Further complicating, is the fact that the ubiquitous subsurface genera, individually, represent a very small proportion of the planktonic community and would not be expected to travel far. In a field transport experiment performed using bacterial strains selected for their adhesion deficiency, the concentration of the bacteria in the water diminished by an order of magnitude across a distance of 7 m (Mailloux, 2003). It is difficult to imagine how a minority population immersed in fracture water at low concentrations (~10 cells/mL) could maintain that concentration over a 500 km distance. Even more challenging is the fact that the isotopic compositions of the fracture water from Dr5IPC, TT109 and NO14 are distinct from those of Be326, MM5 and FI88 (Lau et al., 2014) indicating that the fracture waters have not mixed with each other. In order for the same taxon to be present in isotopically distinct fracture water, it would have to be motile and moving more rapidly than the groundwater flow velocities. The high proportion of shared genera, therefore, implies that shared microorganisms have to be capable of high dispersal through a wide range of subsurface environments. Alternatively, should these ubiquitous subsurface genera of low abundance represent the sessile community that were sloughed off the rock surface by the water current during sampling, the sessile communities are highly homogenous in the subsurface.

Although many of the organisms are shared across subsurface sites, weighted Unifrac significance tests, which take into account the relative abundance of individual phylotypes, showed that all sites are significantly different from one another. This is consistent with the subsurface microbial communities exploiting niches and forming spatially distinctive communities similar to those observed in thermal springs. These changes in the relative abundance of various microorganisms are likely to be tied to the differences in geochemistry or geographic distance. Fuhrman and Steele (2008) and Brazelton et al. (2013) have both reported on the succession of microbes within changing environments through the community sequencing of a time series dataset. As in Brazelton et al. (2013), a pair-wise Pearson correlation analysis between observed subsurface phyla and environmental factors was performed. Although we did not identify any significant correlations between pairwise-comparisons of individual phyla and individual environmental parameters, a Euclidean distance matrix constructed from the scaled values of surface elevation, pH, TDS and the concentration of O_2_, Ca^+^, DIC, CH_4_, and Cl^−^ was highly correlated (*R*^2^ = 0.83) to the subsurface Morisita dissimilarity matrix reported in Figure 5D. The geographical distance matrix did not correlate as well with the subsurface Morisita dissimilarity matrix (*R*^2^ = 0.12). Notably, the Dr5IPC and TT109 microbial communities, separated by 4 km, are more similar to each other (MDI: 0.15) than others yet the microbial communities of NO14 and MM519540, separated by 330 km, are the most similar pair of sites in subsurface dataset (MDI: 0.04). A similar result is observed in the Limpopo thermal springs where the geographical distance matrix was even less correlated to the Morisita dissimilarity matrix (*R*^2^ = 0.03). These results suggest that geochemistry, rather distance, is the most important factor in shaping a subsurface microbial community.

### Comments on experimental design

As our results are entirely based on the analysis of high-throughput sequence data, there are several technical variables that should not be dismissed when evaluating whether or not thermal springs should be used as windows to the subsurface. Firstly, it has been well reported that different primer sets and sequencing technologies result in different taxonomic profiles (Liu et al., 2008; Petrosino et al., 2009; Kumar et al., 2011) and, unfortunately, due to the difficulty in coordinating research in these exotic locations, the same primers and sequencing platform for thermal spring and subsurface datasets were not applied. Additionally, archaea were not included in this current study and, although we have no reason to expect that the majority of the overlap between thermal spring and subsurface communities is within this domain, it is a possibility that we cannot exclude. Furthermore, the volume filtered from the thermal spring (2L) is 3 orders of magnitude less than that filtered from the subsurface sites, 2,850–223,118 L (Table 1) and the pore sizes of the filters were different. Although the spatial beta diversity of the subsurface has not been well characterized, such a large volume filtration can be assumed to minimize the local effects that may be reflected in the thermal spring dataset. This community “homogenization” may explain why there are higher levels of similarity between subsurface sites when compared to the thermal spring datasets.

In addition to the increased volume of sample, subsurface datasets contained over 14,000 reads per site, whereas thermal spring datasets had, at most, 794 reads per site. The increase in number of reads collected corresponds to higher percentage of observed genera relative to the expected number of genera to be discovered (based on Chao1 estimates). It is important to note that when using the Sørensen similarity index as a measure of similarity between sites, sequencing depth is very important. In this study, we generated over 1,000 times more reads than previously collected in the subsurface. As a result of this increased sequencing, we observed higher Sørensen indices than previously reported (Gihring et al., 2006; Chehoud, 2011) (Figure 6). These results suggest that there is a much greater similarity among South African subsurface communities than previously considered (Section Dispersion of Subsurface Populations). Under sampling is likely to have contributed to variability among sites as, prior to this study, the subsurface 16S rRNA gene studies obtained sequences through the generation of a clonal library followed by a selection of clones for downstream Sanger sequencing. MacLean et al. (2007) performed a diversity study on an anaerobic biofilm collected from a South African mine, Evander using, in parallel, cloning and a high throughput PhyloChip. The latter identified over 25 times more species (1,596) than what was observed in the clone library and over 15 times more than the derived Chao1 estimate. With this large number of observed species (far exceeding the Chao1 estimate), it is unlikely that any of the clone library's singleton OTUs would be observed if a replicate was performed. Therefore, it is unsurprising that with an increased sequencing depth we observe a higher percentage of shared taxa between the subsurface communities.

Similar sampling effects may have contributed to the low levels of similarity between the thermal springs sampled (Figure 5). All thermal spring samples were dominated by one genus and contained a high percentage of singleton genera (Supplement Figure 9). Using the Chao1 as an estimate of the actual number genera at each spring, we find that the datasets of all springs are between 47 and 68% complete (Table 3). As shared taxa are likely to persist in the low abundance taxa, an increase in sequencing depth may be needed to reveal the true degree of relatedness between sites.

Although these caveats in experimental design may amplify the differences between observed thermal spring and subsurface communities, it is important to note that in a study of Death Valley springs and their groundwater source, 21,794 partial 16S rRNA sequences were generated through pyrotagging and showed absolutely no overlap of the archaeal and bacterial communities in deep groundwater and surficial springs (Thomas et al., 2013). The lack of consensus between springs and the water that feeds them, as reported by Thomas et al. (2013) and this study highlight the fact that we do not fully understand how microbial communities develop in the transition between surface spring and subsurface waters.

## Conclusions

The lack of overlap in the microbial communities observed between the Limpopo thermal springs and South African subsurface sites, despite their physical-chemical similarities, suggest that thermal springs arising from gravity-driven meteoric water flow may not provide the clearest windows to the terrestrial subsurface. The geographic distance between the thermal springs and the subsurface sites and the fact that four of the springs occur within the Karoo Supergroup, where no subsurface samples have been studied, may account for this lack of sequence overlap. However, despite a geographic separation of up to 500 km and a vertical distribution of 1.0 to 3.1 km, the subsurface sites sampled in this study all shared 220 genera and 1,410 V6 sequences. Based on the ubiquity of this core subsurface set of microorganisms, we would expect that the subsurface communities surrounding the Limpopo thermal springs would also contain several of these pervasive subsurface genera.

The differences in the geographical location of the sampling sites, the region of the 16S rRNA gene, and sequencing depth generated from these two environments may have played a role in minimal overlap observed between thermal spring and subsurface communities. Future work should be performed to confirm or reject the low similarity through parallel (same region, same number of reads, replicates) amplicon studies in the subsurface and thermal springs. By sequencing deeper, more shared organisms are likely to be illuminated. To determine the extent of microbial mixing between rising thermal spring water and local descending meteoric water, these investigations should be combined with intensive geochemical and isotopic measurements of the thermal springs. Wherever possible, deep wells and mines located in thermal spring regions should be studied in parallel. Only by doing this will we be able to decidedly confirm whether or not thermal springs and the microbial communities they contain provide portals into the subsurface biosphere.

### Conflict of interest statement

The authors declare that the research was conducted in the absence of any commercial or financial relationships that could be construed as a potential conflict of interest.

## Supplementary material

The Supplementary Material for this article can be found online at: http://www.frontiersin.org/journal/10.3389/fmicb.2014.00679/abstract. Sequences for the subsurface study have been made available on http://www.ncbi.nlm.nih.gov/, Bio Project ID PRJNA263371. The Limpopo thermal spring sequences are included in the attached.zip file.
