# Supplementary material for: Comparisons of the composition and biogeographic distribution of the bacterial communities occupying South African thermal springs with those inhabiting deep subsurface fracture water
Source: Front Microbiol. 2014 Dec 17;5:679. doi: 10.3389/fmicb.2014.00679 (PMC4269199; doi:10.3389/fmicb.2014.00679)
Supplement: Supplementary file 1 [file Presentation1.PDF]

Supplement for: Comparisons of the composition and biogeographic distribution of the bacterial communities occupying South African thermal springs with those inhabiting deep subsurface fracture water

C. Magnabosco[1]*, M. Tekere[2], M.C.Y. Lau[1], B. Linage[3], O. Kuloyo[3], M. Erasmus[3], E. Cason[3], E. van Heerden[3], G. Borgonie[4], T.L. Kieft[5], J. Olivier[2], T.C. Onstott[1]

[1]Department of Geosciences, Princeton University, Princeton, NJ, USA
[2]Department of Environmental Sciences, School of Agriculture and Environmental Sciences, University of South Africa, Florida, South Africa
[3]Metagenomics Platform, Department of Microbial, Biochemical and Food Biotechnology, University of the Free State, Bloemfontein, South Africa
[4]Ghent University, Ghent, Belgium
[5]Biology Department, New Mexico Tech, Socorro, NM, USA

***Correspondence:** Cara Magnabosco, Department of Geosciences, Guyot Hall, Princeton University, Princeton, NJ, 08544, USA.
cm13@princeton.edu

Supplement Table 1. Additional Sampling Information

Parameter	Be326_2011	Be326_2012	Dr5IPC	FI88	MM51940	NO14	TT109_Bh2
Cells/mL¶	1.26×10^3	3.4×10^4	8.04×10^2	2.8×10^3	2.2×10^2	6.6×10^2	3.3×10^4*
Volume Filtered (L)	4,875	10,214	12,005	223,118	11,362	2,850	26,809
Estimated Biomass^ (mg)	0.18	6.6	0.18	12	0.047	0.03	16.8

¶To perform cell counts, 45mL of fracture water was fixed with filtered formaldehyde (final concentration, 4% v/v). The total sample was then filtered onto a 0.2 μm polycarbonate membrane filter, stained with DAPI, and counted under an epifluorescent microscope.
* Phospholipid Fatty Acid (PLFA) estimate (Simkus et al., *in prep*)
^Biomass calculated as #cells/mL x 1.9×10^{-14}g/cell x 1000 x Volume filtered (L). The g/cell is from Onstott *et al.*, (2014).

Supplement Table 2. Contaminating Sequences Identified by Hilary Morrison, MBL (hmorrison1981@gmail.com)

Acinetobacter:

> Acinetobacter_1

TGGTCTTGACATAGTAAGAACTTTCCAGAGATGGATTGGTGCCTTCGGGAACTTACATAC

> Acinetobacter_2

TGGCCTTGACATAGTAAGAACTTTCCAGAGATGGATTGGTGCCTTCGGGAACTTACATAC

> Acinetobacter_3

TGGCCTTGACATACTAGAAACTTTCCAGAGATGGATTGGTGCCTTCGGGAATCTAGATAC

> Acinetobacter_4

TGGTCTTGACATACTAGAAACTTTCCAGAGATGGATTGGTGCCTTCGGGAATCTAGATAC

OD1:

>OD1_1

AGGGCTTGAAAGACTAGTGAAAGTCTTCTGAAAAGAAGATCTACCCACAAGGACACTAGTTC

>OD1_2

AGGGCTTGAAAGACTAGTGAAAGTCTTCTGAAAAGAAGGCCTACCCACAAGGACACTAGTTC

>OD1_3

AGGGCTTGAAAGACTAGTGAAAGTCTTCTGAAAAGAAGACCTACCCACAAGGACACTAGTTC

>OD1_4

AGGGCTTGACAGACTAGTGAAAATCTGCCAAAAGGCAGATCTACCCACAAGGACACTAGTCC

>OD1_5

AGGGCTTGAAAGACTAGTGAAAGTCTTCTGAAAAGAGGACCTACCCACAAGGACACTAGTTC

Supplement Table 3. Number of Genera and OTUs observed in subsurface

Site	Genera Observed by GAST	# OTUs at 97% (SLP-PWAL)	# OTUs @ 97% (UPARSE)
Beatrix Au Mine - BE326FW250111Bh2	551(332)	5952(2,478)	6372(3,916)
Beatrix Au Mine - BE326FW270712Bh2	634(434)	8900(3,987)	8857(4,435)
Driefontein Au Mine - Dr5IPCFW280711	549(353)	7589(3,059)	7596(4,654)
Finsch Diamond Mine - FI88FW031012	573(355)	7422(3,053)	7321(4,579)
Masimong Au Mine - MM51940FW200712	655(427)	9456(3,786)	9712(5,335)
Zondereinde Pt Mine - NO14FW130912	640(428)	8917(3,774)	9328(5,312)
Tau Tona Au Mine - TT109FW060311Bh2	569(373)	7741(3,316)	7919(4,837)

() Totals excluding singleton sequences

Supplement Table 4. Diversity Statistics after removal of singletons (for comparison to Table 3 in main text)

Site	Number of Reads	Number of reads classified by RDP* or GAST^	Number of Genera Observed	Pielou's Evenness	ACE[a]
Beatrix Au Mine - BE326FW250111Bh2	14,227	7,238 (51%)	332	0.54	332±7
Beatrix Au Mine - BE326FW270712Bh2	23,343	12,302 (53%)	434	0.51	434±9
Driefontein Au Mine - Dr5IPCFW280711	18,215	8,233 (45%)	353	0.48	353±7
Finsch Diamond Mine - FI88FW031012	18,719	9,225 (49%)	355	0.51	355±8
Masimong Au Mine - MM51940FW200712	22,984	10,894 (47%)	427	0.52	427±9
Zondereinde Pt Mine - NO14FW130912	22,237	11,304 (51%)	428	0.54	428±9
Tau Tona Au Mine - TT109FW060311Bh2	19,367	9,973 (51%)	373	0.51	373±8

[a]ACE is used instead of Chao1 as there are no singletons. ACE is calculated by Chao & Lee (1992)
Chao A, Lee S-M. Estimating the number of classes via sample coverage. J Am Stat Assoc.1992;87:210–217.

Supplement Figure 1.

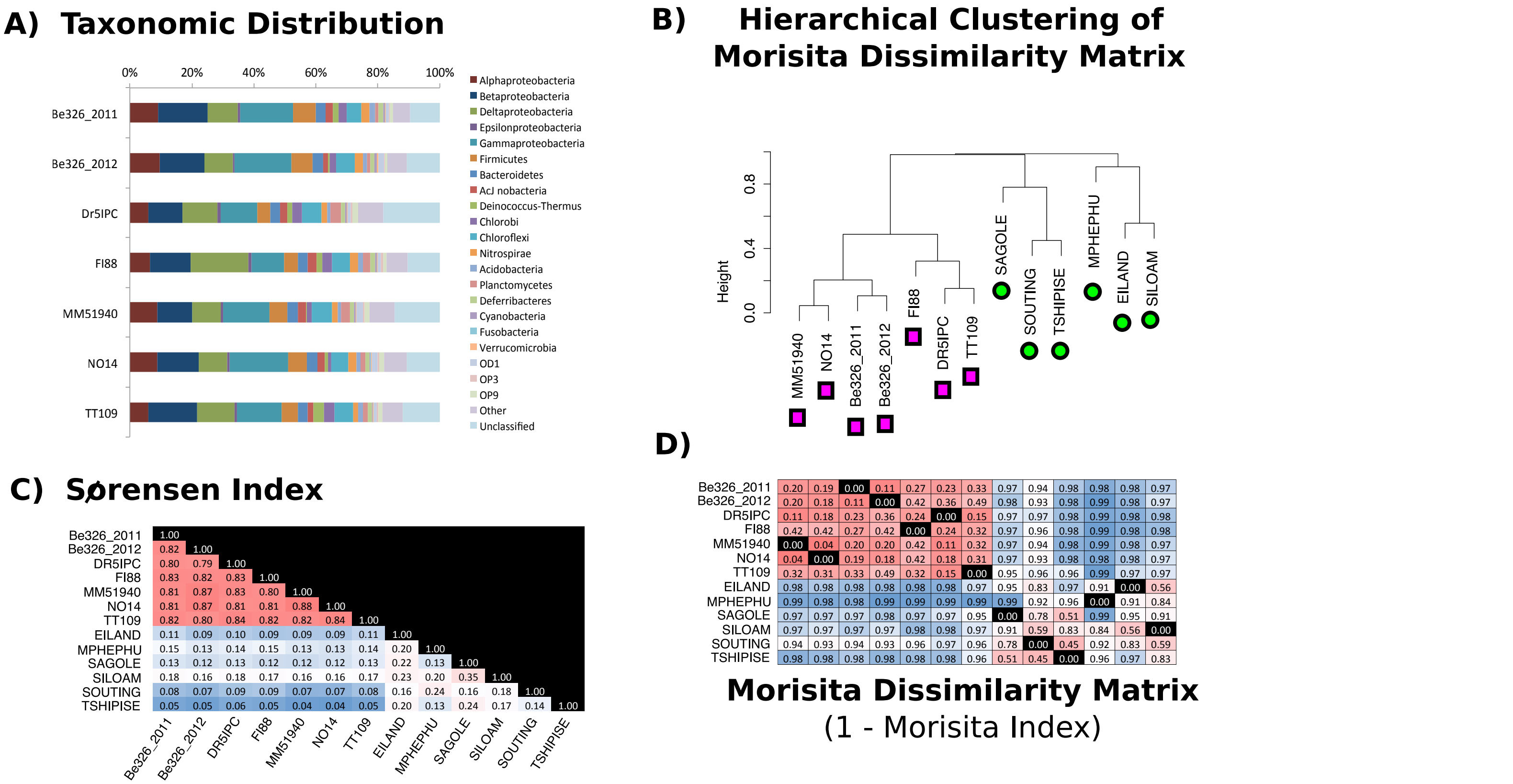

Figure 1a shows the taxonomic distribution of the dataset when singletons are removed. Sørensen similarity index of non-singleton genera are shown in (b) and Morisita dissimilarity indices (1-Morisita index) are shown in (d). Color-coding of b,d are purely for visual aid where red indicates more similar pairs and blue is indicative of more distant pairs. Figure c is a visual representation of the hierarchical clustering of the Morisita dissimilarity matrix (d). Sites in (c) are labeled with pink squares for subsurface samples and green circles for thermal spring samples. The columns of d are indicated by the branches of (c) whereas the columns of b are labeled. Black squares labeled 0.00 in (d) indicate same sample in c's branch as the row.

Supplement Figure 2.

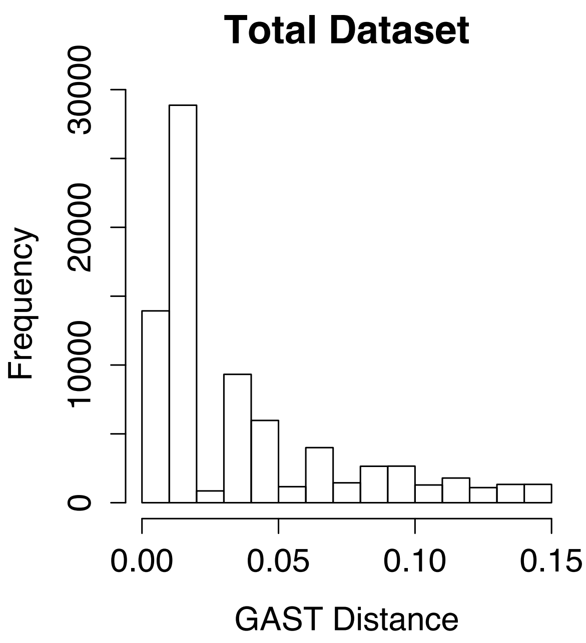

A histogram of GAST distances for the total dataset (left) and when singletons are removed (right). The GAST distance is shown on the x-axis while the number of sequences contributing to each distance is the height of the bar.

Supplement Figure 3. Geographic location of sample sites.

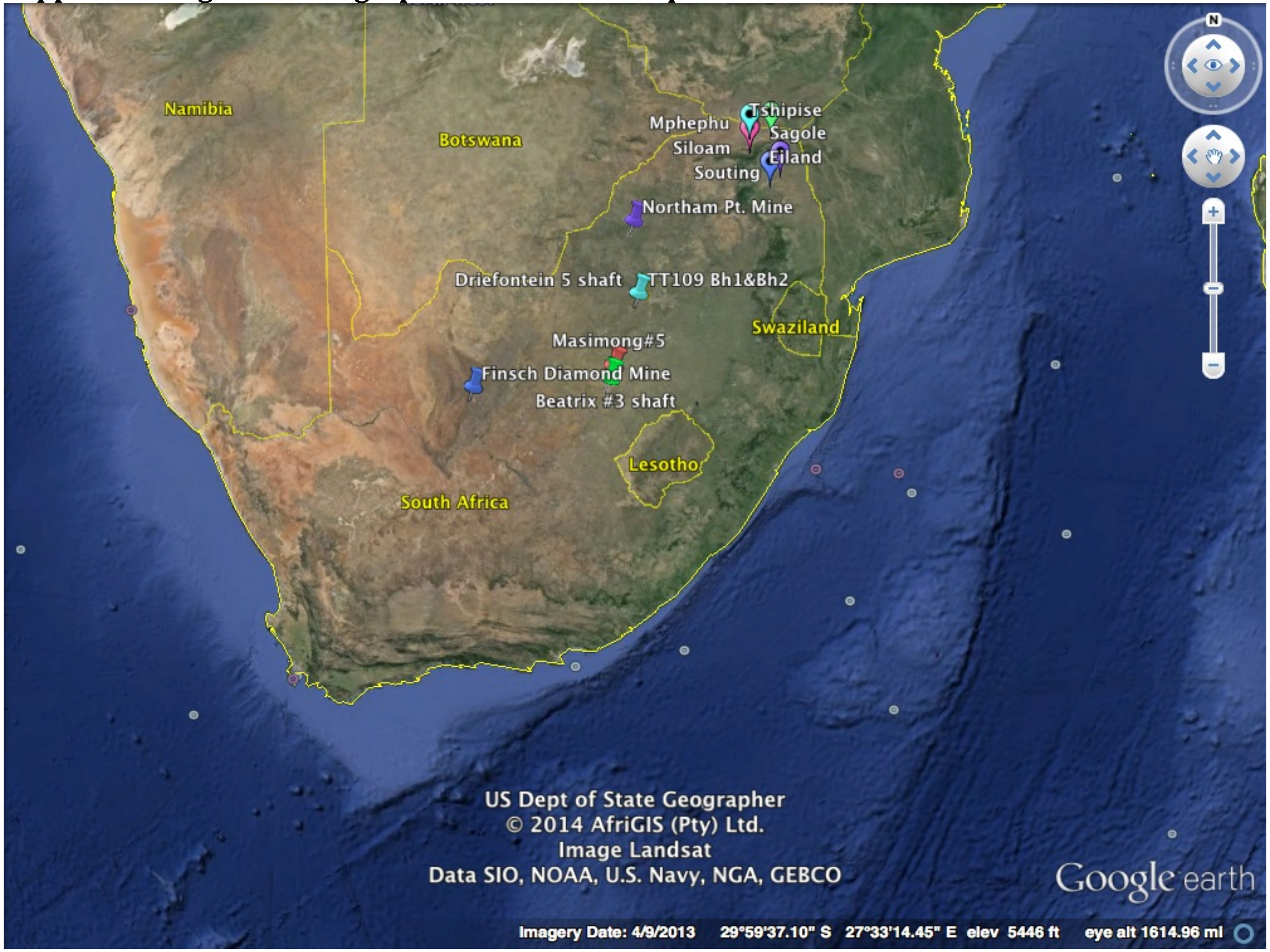

A map of where thermal springs (teardrops) and subsurface (pins) samples were collected.

Supplement Figure 4. Differences in length of thermal spring amplicons

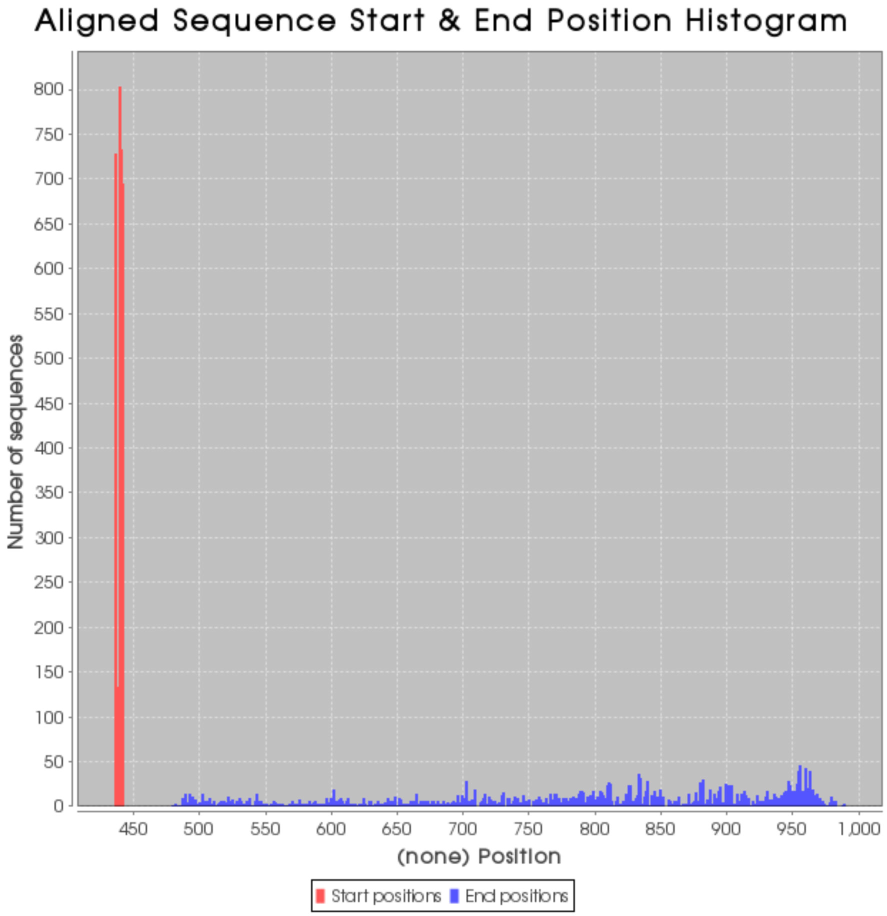

This figure is generated in the RDP Pipeline Tools alignment. It is a histogram that represents the start and end positions of sequences on a histogram. The start position is in red while the end position is in blue. The x-axis represents the position in the alignment while the y-axis is the number of sequences with that start/end position.

Supplement Figure 5 Rarefaction Curves

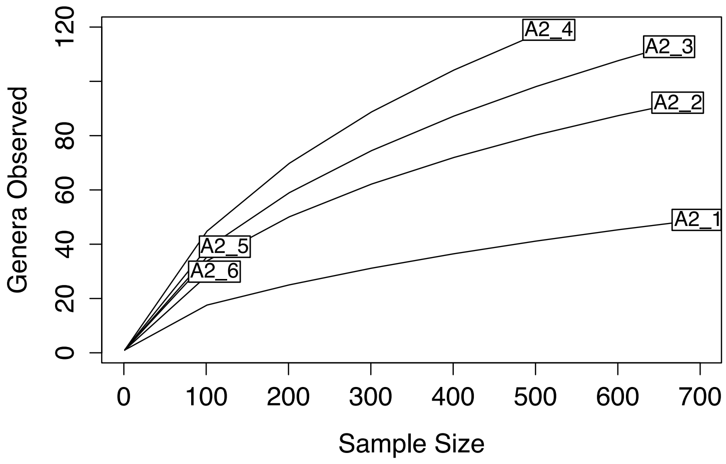

Rarefaction curve of thermal spring dataset. A2_1 = Eiland, A2_2 = Mphephu, A2_3 = Sagole, A2_4 = Siloam, A2_5 = Souting, A2_6 Tshipise

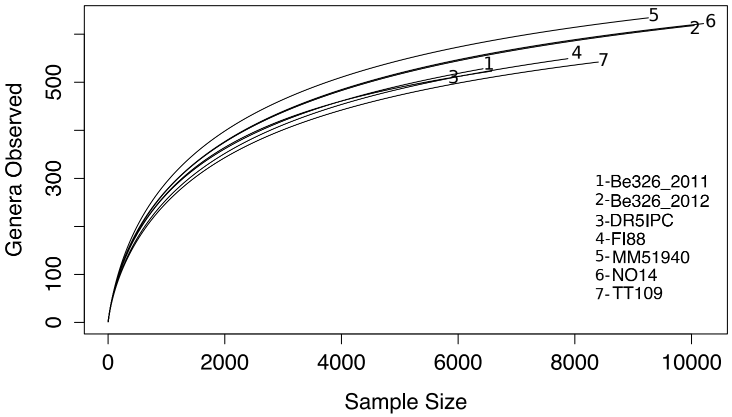

Rarefaction curve of subsurface data.

Supplement Figure 6. Unweighted Pairwise UniFrac significance of subsurface dataset

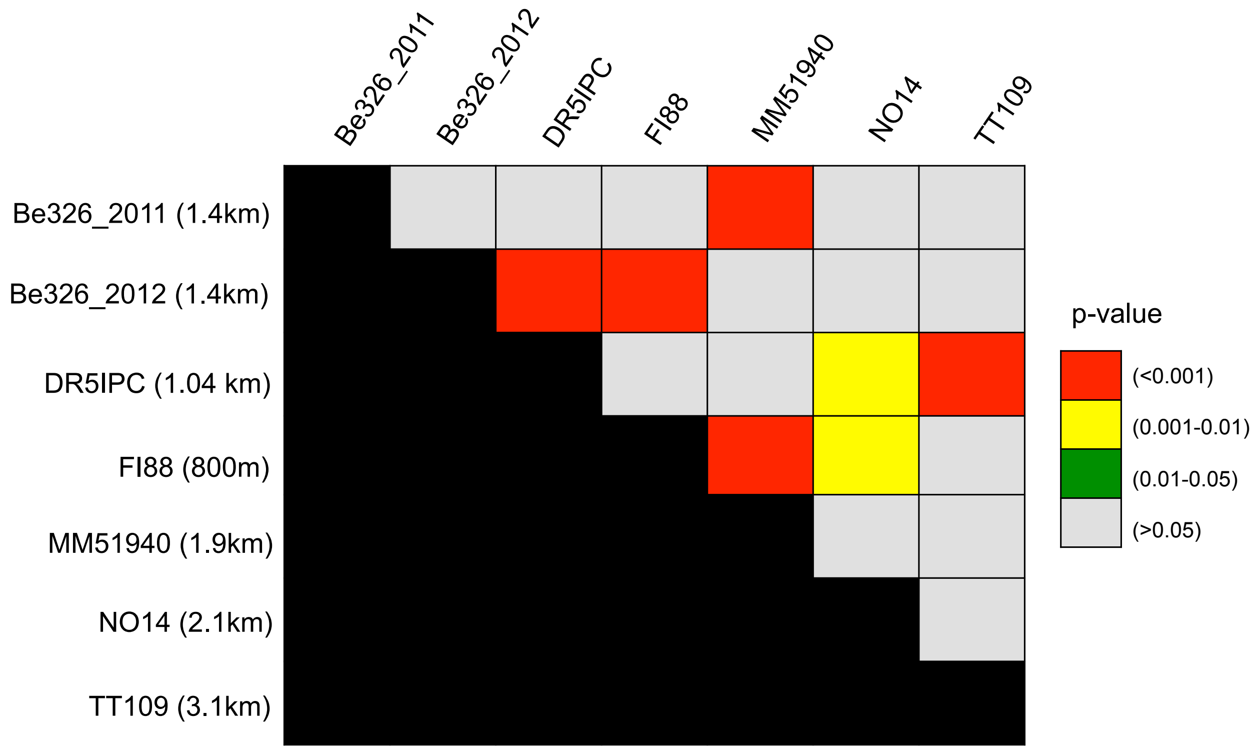

The results of unweighted pairwise UniFrac significance test. Sites are listed in columns and rows and significance is given by color.

Supplement Figure 7. Relative abundance of the 1,410 sequences identified in all subsurface sites

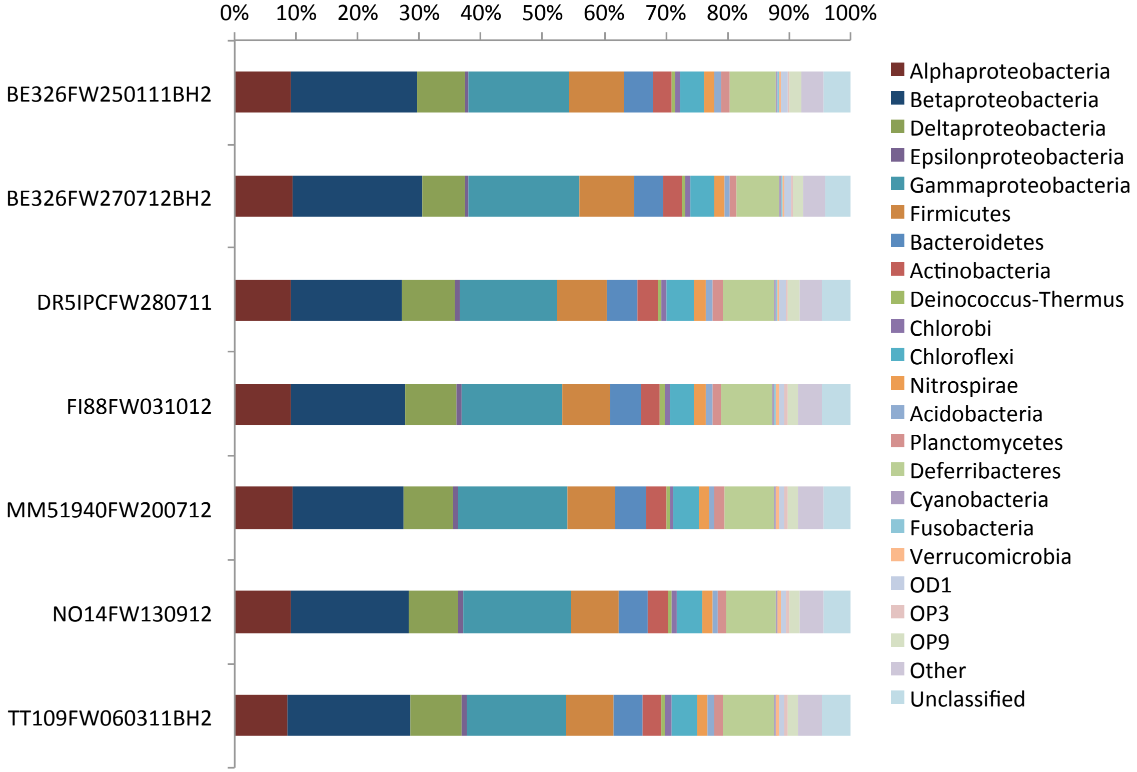

Supplement Figure 8. Shared Sequences in Subsurface and Thermal Springs
A) Number of mismatches=0
*A2_1 = Eiland, A2_2 = Mphephu, A2_3 = Sagole, A2_4 = Siloam, A2_5 = Souting, A2_6 Tshipise

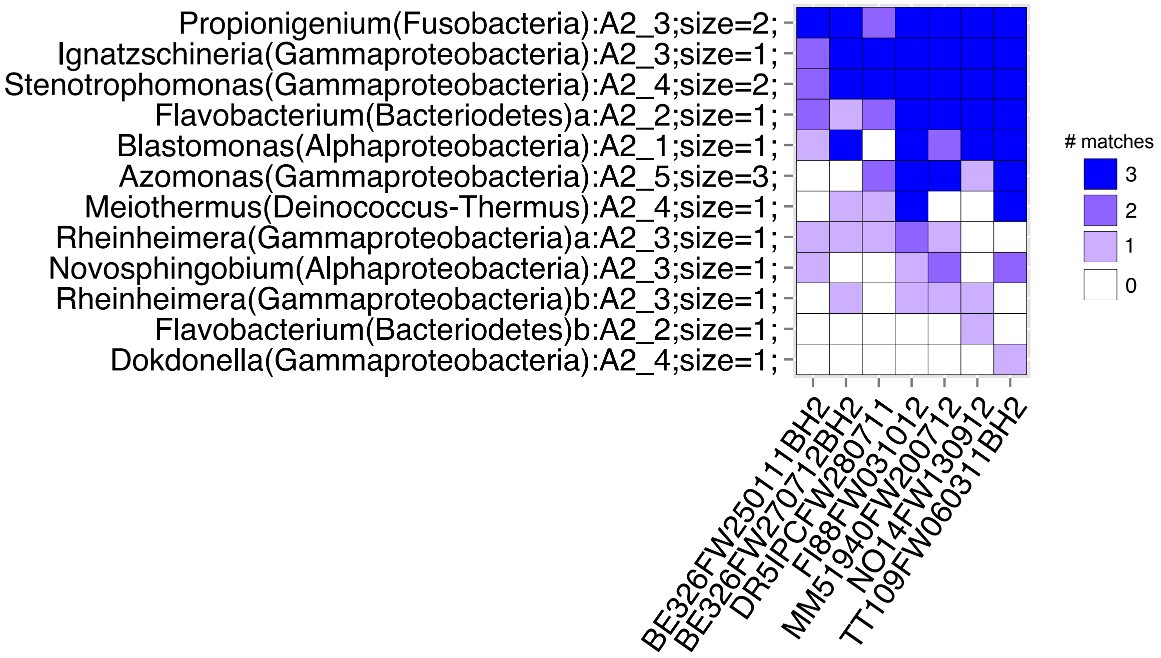

Sequences shared between thermal spring and subsurface datasets when no mismatches are allowed in Bowtie. The description of the shared sequence is given in the row label, followed by the thermal spring the sequence was found in, and the number of sequences identified in the thermal spring dataset. The corresponding number of sequences identified is given by the color and the sample site is given by the column.

B) Number of Mistmatches=2

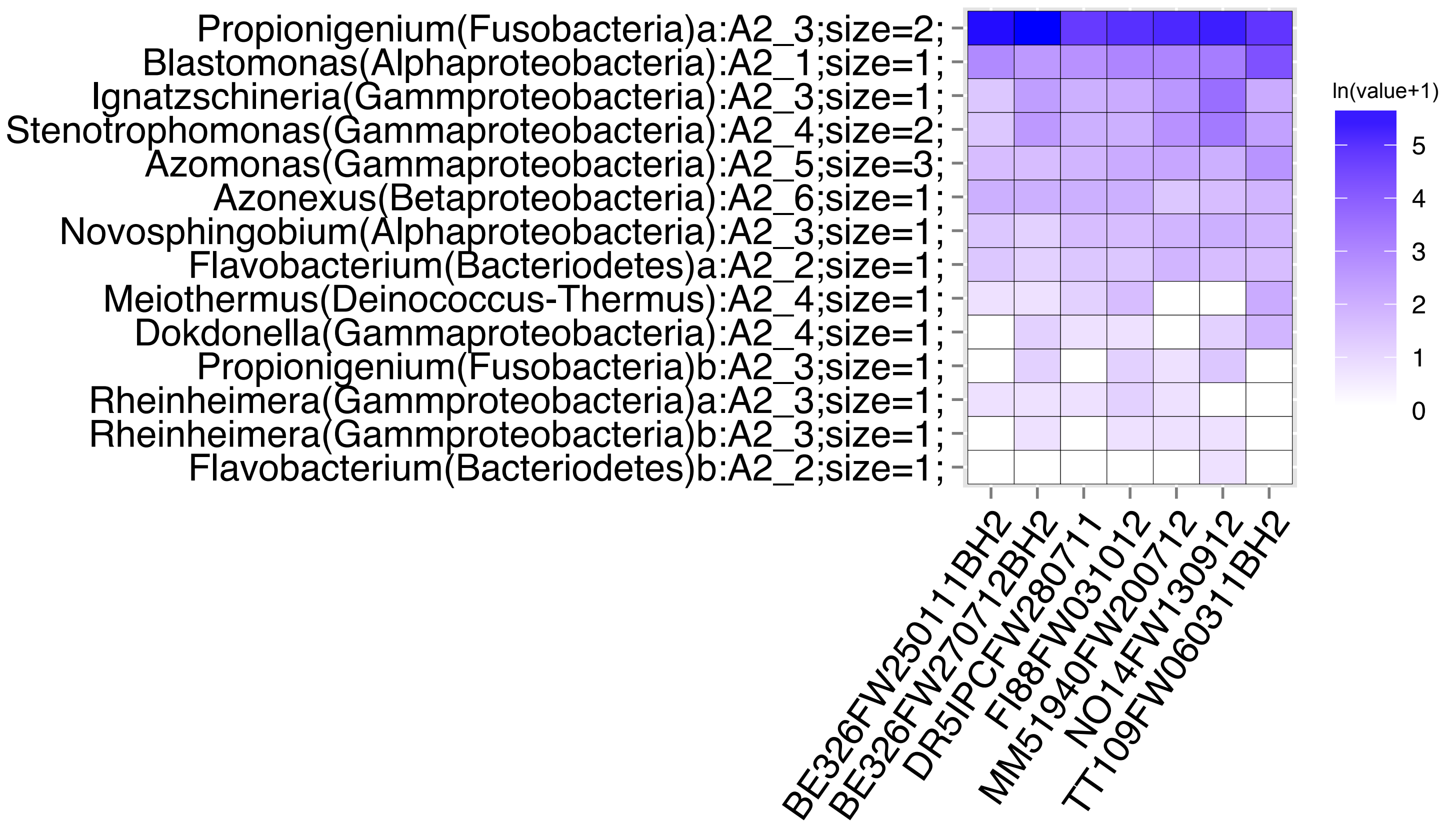

Sequences shared between thermal spring and subsurface datasets when 2 mismatches are allowed in Bowtie. The description of the shared sequence is given in the row label, followed by the thermal spring the sequence was found in, and the number of sequences identified in the thermal spring dataset. The corresponding natural log of the number of sequences identified is given by the color and the sample site is given by the column.

Supplement Figure 10. Histograms of thermal spring genus-abundances

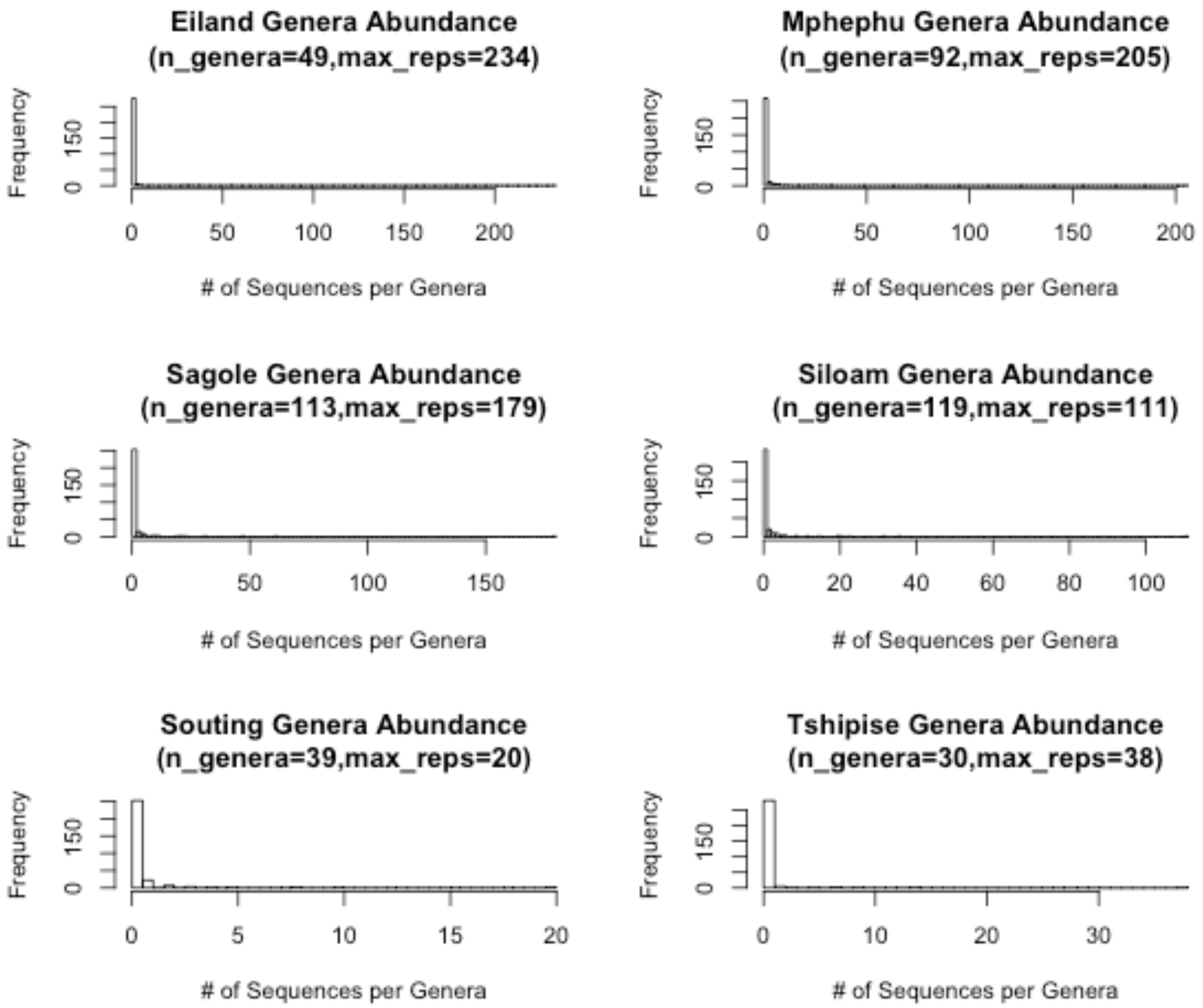

Histograms of the number of sequences per genera (x-axis) are show in the figure above. Here, "n_genera" indicates the number of genera per site and "max_reps" indicates the number of sequences contributing to the largest genus at each site.
